# Genetic Determinants of Gating Functions: Do We Get Closer to Understanding Schizophrenia Etiopathogenesis?

**DOI:** 10.3389/fpsyt.2020.550225

**Published:** 2020-11-25

**Authors:** Rastislav Rovný, Dominika Besterciová, Igor Riečanský

**Affiliations:** ^1^Department of Behavioural Neuroscience, Institute of Normal and Pathological Physiology, Centre of Experimental Medicine, Slovak Academy of Sciences, Bratislava, Slovakia; ^2^Social, Cognitive and Affective Neuroscience Unit, Department of Cognition, Emotion, and Methods in Psychology, Faculty of Psychology, University of Vienna, Vienna, Austria

**Keywords:** schizophrenia, endophenotypes, intermediate phenotype, prepulse inhibition, P50, sensory gating, sensorimotor gating, startle reflex

## Abstract

Deficits in the gating of sensory stimuli, i.e., the ability to suppress the processing of irrelevant sensory input, are considered to play an important role in the pathogenesis of several neuropsychiatric disorders, in particular schizophrenia. Gating is disrupted both in schizophrenia patients and their unaffected relatives, suggesting that gating deficit may represent a biomarker associated with a genetic liability to the disorder. To assess the strength of the evidence for the etiopathogenetic links between genetic variation, gating efficiency, and schizophrenia, we carried out a systematic review of human genetic association studies of sensory gating (suppression of the P50 component of the auditory event-related brain potential) and sensorimotor gating (prepulse inhibition of the acoustic startle response). Sixty-three full-text articles met the eligibility criteria for inclusion in the review. In total, 117 genetic variants were reported to be associated with gating functions: 33 variants for sensory gating, 80 variants for sensorimotor gating, and four variants for both sensory and sensorimotor gating. However, only five of these associations (four for prepulse inhibition—CHRNA3 rs1317286, COMT rs4680, HTR2A rs6311, and TCF4 rs9960767, and one for P50 suppression—CHRNA7 rs67158670) were consistently replicated in independent samples. Although these variants and genes were all implicated in schizophrenia in research studies, only two polymorphisms (*HTR2A* rs6311 and *TCF4* rs9960767) were also reported to be associated with schizophrenia at a meta-analytic or genome-wide level of evidence. Thus, although gating is widely considered as an important endophenotype of schizophrenia, these findings demonstrate that evidence for a common genetic etiology of impaired gating functions and schizophrenia is yet unsatisfactory, warranting further studies in this field.

## Introduction

Sensory and sensorimotor gating are conceptualized as basic cognitive processes that regulate the processing of sensory input by the brain. It has been suggested that gating represents a filtering mechanism, preventing distraction and sensory overload, or a protective mechanism, securing uninterrupted processing of stimuli (1–4). Importantly, it has been further postulated that disrupted gating may contribute to information processing deficits, cognitive fragmentation, and thought disorder, the hallmark feature of schizophrenia psychosis (5–8).

Sensory gating is routinely examined by measuring the electroencephalographic event-related potentials (ERPs) during a paired-pulse paradigm (7). The paradigm comprises trials with two identical auditory stimuli of the same intensity, a conditioning stimulus (S1) and a testing stimulus (S2), that are presented successively with an interstimulus interval of 500 ms (9, 10). Auditory stimuli elicit an ERP, which is characterized by a positive peak ~40–90 ms after stimulus onset, known as P50 wave. It has been suggested that response to S1 triggers an inhibitory mechanism that results in a reduced amplitude of the P50 wave after the presentation of S2. The diminution of the P50 wave to S2 relative to that elicited by S1, called P50 suppression or P50 gating, is the operational definition of sensory gating (7, 10, 11). Other well-established, but less commonly assessed, measures of sensory gating include the suppression of the N100 and P200 ERP waves (12, 13). The most widely used measure of sensorimotor gating, on the other hand, is prepulse inhibition (PPI) of the acoustic startle reflex. During the PPI paradigm, the presentation of a sudden and intense auditory startling stimulus (pulse) is preceded (usually 30–120 ms) by a weaker non-startling stimulus (prepulse). This leads to a reduction in the startle reflex also known as PPI. In humans, PPI is commonly quantified by measuring the eye-blink component of the startle reflex using electromyography of the periocular muscles (14–16).

Both PPI and P50 gating are robustly reduced in schizophrenia spectrum disorders [e.g., (17–20)], but also several other psychiatric conditions, in particular, bipolar disorder and obsessive–compulsive disorder [e.g., (21–28), for review see e.g., (29, 30)]. Deficits in PPI and P50 gating were reported not only in psychiatric patients but also in their unaffected first-degree relatives [(19, 31–33), for a recent review of PPI studies see ref. (34)]. Several studies have demonstrated a significant heritability of these measures, ranging 29–58% for PPI and 10–68% for P50 gating (31, 35–42). Given these attributes, including a high test–retest reliability, PPI and P50 suppression deficits are considered as important endophenotypes of neuropsychiatric disorders (20, 37), i.e., intermediate phenotypes (or markers) that are associated with disorders but are simpler in terms of the genetic and neurobiological architecture (43–45). Endophenotypes represent an important approach to deal with the complexity and polygenic nature of mental disorders such as schizophrenia. It is supposed that studying the genetic architecture of endophenotypes and their relationship with biological processes impaired in neuropsychiatric disorders may contribute to a better understanding of the underlying pathophysiology [e.g., (46)]. The genetic basis of gating in humans has been intensively studied over the last decades, and it has become apparent that a significant genetic component is involved in both PPI and P50 suppression. Despite an extensive and rapidly growing body of literature on the relationship between genotype and gating in humans, the underlying genetic architecture of these endophenotypes remains elusive due to fragmentary evidence and lack of verification. Recently, Quednow et al. (47) carried out a systematic review (and a meta-analysis) of human association studies of PPI (sensorimotor gating). However, a similar assessment of sensory gating studies is lacking, as is an integrative review of genetic determinants of both sensory and sensorimotor gating functions. The aim of this work was thus to evaluate current knowledge regarding the etiopathogenetic links between genetic variation, gating efficiency, and schizophrenia. For this purpose, we carried out a systematic review of published genetic association studies assessing the relationship between genetic variation and the efficiency of sensory and sensorimotor gating in humans. Furthermore, we critically assessed the reliability of these findings by examining the quality of the studies, the number of replications, and the relative number of positive and negative results. Finally, we evaluated the evidence for genetic mechanisms shared between gating and schizophrenia.

## Materials and Methods

### Study Design

The review process followed the Preferred Reporting Items for Systematic Reviews and Meta-Analyses (PRISMA) guidelines (48). The Covidence online software (Covidence systematic review software, Veritas Health Innovation, Melbourne, Australia; available at www.covidence.org) was used to facilitate the process of screening, paper selection, and data extraction.

### Search Strategy

To identify eligible studies, we performed a systematic, comprehensive search of the published literature using Pubmed and Scopus until October 2019. The electronic databases were searched using the combination of Boolean operators and the following key words: sensorimotor gating, sensory gating, prepulse inhibition, P50, startle, polymorphism, gene and human, among others (for exact search phrases utilized, see Supplementary Data 1). Also, a secondary search of relevant articles was performed by screening the references of included full-text papers.

### Inclusion Criteria and Study Selection

To be included in the review a study had to meet the following inclusion criteria: (1) study design of a candidate gene association study (CGAS), genome-wide association study (GWAS), or included these studies as a part of more complex study design (e.g., pharmacogenetic study), (2) study enrolled human subjects (healthy participants or psychiatric patients), (3) study outcomes included sensorimotor gating as indexed by PPI or sensory gating indices such as P50, N100, or P200 suppression, and (4) the report was written in English. Only original research papers were included; other article types, such as reviews, meta-analyses, case reports, editorials, and commentaries, were excluded.

The search results were imported to the Covidence, and after removing duplicates, titles and abstracts of identified studies were independently screened by the first and the second author (RR and DB). At this stage, only irrelevant studies that obviously did not meet the inclusion criteria were excluded from the review. Next, for the remaining potentially eligible papers, the same two authors independently assessed full texts to select only those articles that meet all of the abovementioned inclusion criteria. Any disagreement between the two reviewers was discussed and resolved by consensus. If needed, the third author (IR) was involved to reach a decision.

### Data Extraction

The following information was extracted from the selected studies: name of the first author, affiliation of the first author, year of publication, country, study design, sample characteristics (sample size, mean age, sex ratio, race/ethnicity, inclusion/exclusion criteria and population—healthy vs. psychiatric patients), parameters of the auditory stimulation (duration and intensity of acoustic stimuli, background sound intensity, and sound frequency), electrode placement, statistical test used, description of assessed polymorphisms (polymorphism type, reference number/label, chromosomal position, closest gene, reference and minor allele frequency, functional consequence, and association with disorders), and study outcomes of interest (genotype effects on PPI and sensory gating across all samples including *p*-value, effect size, direction of the effect, mean values, and standard deviations). The data extraction was carried out by DB and RR, working independently and in duplicate, using the Covidence data extraction tool. All data extraction forms from both reviewers were inspected for potential errors and compiled by RR.

### Quality Assessment

The quality of genetic studies (Q-Genie) 11-item tool was used to evaluate the quality of all included studies. We opted for this tool since it was specifically developed and validated to facilitate the assessment of the global quality of genetic association studies, and it proved to be valid and reliable for both expert and non-expert raters (49). The quality assessment was conducted by DB and RR, working independently and in duplicate. The final quality score for each study was calculated by averaging the respective scores from the two reviewers. Following the Q-Genie scoring system, scores below 33 indicate poor-quality studies, scores between 33 and 40 indicate studies of moderate quality, and scores above 40 indicate good-quality studies. The degree of interobserver agreement was tested using the Cohen's kappa coefficient (κ), with values categorized as poor (≤0.20), fair (0.21–0.40), moderate (0.41–0.60), substantial (0.61–0.80), and almost perfect agreement (>0.80) (50). The Cohen's kappa coefficient was computed by using the FREQ procedure in SAS Studio software (SAS University Edition, release 3.8, SAS Institute, Cary, NC, USA).

## Results

### Identification of Relevant Studies

**Figure 1:**
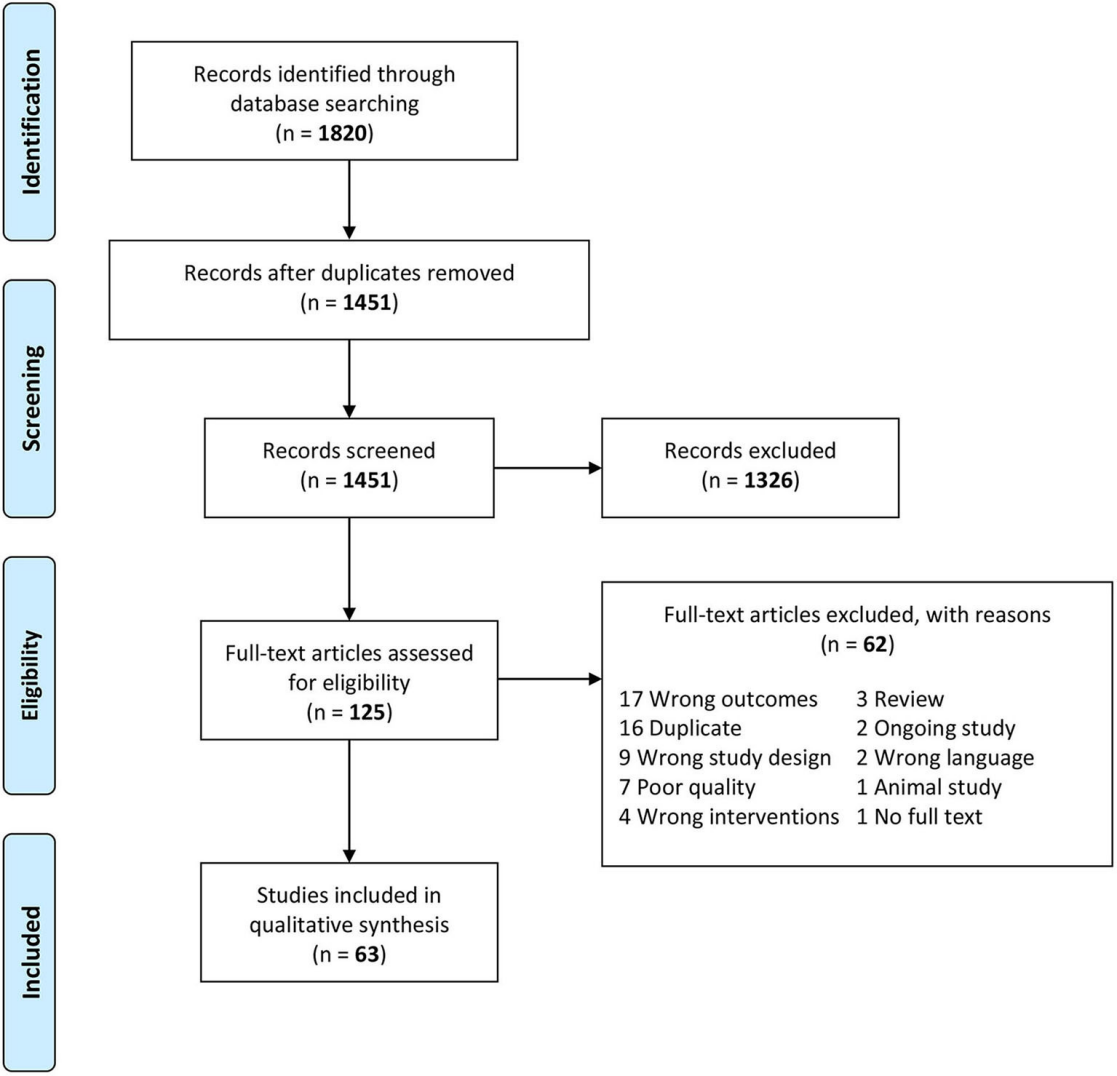
The Preferred Reporting Items for Systematic Reviews and Meta-Analyses (PRISMA) flow diagram of the study selection process (51).

The systematic search yielded 1,820 potentially relevant references. After removing 369 duplicates and 1,326 irrelevant articles identified by screening abstracts, the full texts of the remaining 125 papers were assessed for eligibility. Of them, excluded were 39 studies that did not meet the inclusion criteria and 16 duplicates not captured by Covidence, leaving 70 papers. Based on the Q-Genie scoring system, 41 out of the 70 relevant studies (58.6%) were rated high quality, 22 (31.4%) moderate, and 7 (10.0%) poor. A weighted kappa value of 0.55, 95% CI (0.50–0.60) indicates a moderate agreement between the two raters (DB and RR). Poor-quality studies were excluded from the systematic review due to concerns about the validity of results, leaving 63 eligible papers. No additional papers were identified by screening the references of the included articles. The process of study selection is depicted in the PRISMA flow diagram below (Figure 1).

### Basic Description of the Included Studies

The final selection included 53 CGAS, 3 GWAS, and seven pharmacogenetic studies (25, 52–113). These studies investigated in total 63 independent sample groups: 36 samples of healthy individuals, 20 patient samples (16 with schizophrenia), and seven samples involving both patients and healthy individuals. Sensorimotor gating (PPI) was assessed in 41 studies, sensory gating (P50 or N100 suppression, for simplicity thereafter referred to as P50 gating) in 18 studies, and four studies assessed both measures. A short summary of the basic characteristics of the studies is provided in Table 1; details for each study included in this review are provided in Supplementary Table 1.

**Table 1:** Overview of the basic characteristics of the studies included in the review (25, 52–113).

		**No. of samples**				**Sample size**			**Gating measure**		
**Study design**	**No. of studies**	**Total**	**Healthy**	**Patients (SZ)**	**Mixed**	**Mean**	**SD**	**Range**	**PPI**	**P50**	**Both**
CGAS	53	53	28	20 (16)	5	153	244	23–1,821	34	15	4
GWAS	3	4	2	0	2	719	385	306–1,212	2	1	0
Pharmacogenetic	7	6	6	0	0	57	41	23–114	5	2	0
*Total*	63	63	36	20 (16)	7				41	18	4

*CGAS, candidate gene association study; GWAS, genome-wide association study; SZ, patients with schizophrenia; PPI, prepulse inhibition of the acoustic startle reflex; P50, suppression of wave P50 or N100 of the auditory evoked potential*.

### Identification and Description of Genetic Polymorphisms

Data extraction from the eligible studies resulted in the identification of 201 polymorphisms located within or close to 77 genes. Association with PPI was tested for 125 polymorphisms. Among them, 84 variants, within or close to 37 genes, were reported as significantly (*p* < 0.05) associated with PPI in at least one sample. Association with P50 gating was investigated for 109 polymorphisms, of which 37, located within or close to 13 genes, were significantly associated with this measure in at least one sample. Association with both PPI and P50 gating was investigated in 54 variants and a significant association with both measures was reported for four polymorphisms (*COMT* rs4680, rs165599, *ANKK1* rs1800497, and *TCF4* rs9960767). A vast majority of the variants were single nucleotide polymorphisms (SNPs, Table 2; for a detailed summary see Supplementary Tables 2–5).

**Table 2:** Overview of the reported associations with gating measures.

		**Reported associations**		**Positive associations**^c^		
**Gating measure**	**No. of investigated variants^a^**	**Sig.^b^**	**Nonsig**.	**SNP**	**NonSNP**	**Genes**
PPI	125	84 (3)	100	65	19	37
P50	109	37 (9)	85	30	7	13

a *Includes only variants investigated in candidate gene association studies and pharmacogenetic studies*.

b *Associations reported as significant (p < 0.05), number in parentheses: no. of significant associations reported in genome-wide association studies*.

c *No. of variants/genes positively associated with gating function in at least one study. PPI, prepulse inhibition of the acoustic startle reflex; P50, suppression of wave P50 or N100 of the auditory evoked potential; SNP, single nucleotide polymorphisms; non-SNP, includes copy number variants, combined genotype, haplotypes, genetic interaction, indels, short tandem repeats*.

To provide insight into the involved biological mechanism, we conducted an enrichment analysis using the Gene Ontology Resource (114–116). The associated variants were annotated by dbSNP and clustered based on the overrepresentation of the corresponding genes in the Gene Ontology classification section Biological Processes. The results of this analysis are provided in Table 3. Associations were considered as consistent (reliable) if a significant association with PPI or P50 gating was reported in at least two independent samples, and the number of reported significant associations was higher than the number of null findings. For both PPI and P50 gating, the reported positive associations included several genes involved in neurodevelopmental processes and/or cellular signaling (in particular glutamatergic, dopaminergic, serotoninergic, and cholinergic neurotransmission). However, most of the polymorphisms for which positive associations were reported were explored in only one published study (PPI: 64.3%, P50: 81.1%). Applying our criterion of reliability, only four associations with PPI (*CHRNA3* rs1317286, *COMT* rs4680, *HTR2A* rs6311, and *TCF4* rs9960767) and one with P50 gating (*CHRNA7* rs67158670) can be considered as consistent.

**Table 3:** Summary of genes and genetic variants associated with gating functions.

**Gene ontology category (Section biological processes)**	**Genes with positive associations**	**Reliable associations^a^**
Nervous system development	PPI: *AUTS2, AVPR1A, CTNNA2, ERBB4, KCNQ2, NCAM1, NGF, NOS1, NRG1, OXTR, RELN, TCF4, TSPAN2* P50: *DISC1, ERBB4, FLRT2, TCF4*	PPI: *TCF4* rs9960767
Synaptic transmission, glutamatergic	PPI: *GRID2, GRIK3, GRIN2A, GRIN3A, GRIN3B* P50: *GRID2, GRIK4*	
Synaptic transmission, cholinergic	PPI: *CHRNA3, CHRNA4, CHRNA7* P50: *CHRNA7*^c^, *CHRFAM7A*	PPI: *CHRNA3* rs1317286 P50: *CHRFAM7A* rs67158670
GPCR signaling pathway, coupled to cyclic nucleotide second messenger	PPI: DRD2, *DRD3, HTR1A*^c^, *HTR2A* P50: *GRM3*	PPI: *HTR2A* rs6311
Regulation of calcium ion transport	PPI: *CAMK2A, FMR1, NOS1AP* P50: *CACNAC1*	
Neurotransmitter reuptake	PPI: *SLC1A2, SLC6A3* P50: *SLC6A3*	
Dopamine metabolic process	PPI: *COMT, DAO, DBH* P50: *COMT*	PPI: *COMT* rs4680
Serotonin metabolic process	PPI: *TPH2*^b^	
Proline metabolic process	PPI: *PRODH*	
Unclassified	PPI: *ANKK1, KPNA4* P50: *ANKK1*	

a *Criteria of reliability: significant association was reported in at least two independent samples and the number of reported significant associations was higher than number of reported null results*.

b *A significant association only at the level of haplotype, not single polymorphism*.

c *A significant association only at the level of combined genotype of two or more variants. GPCR, G protein-coupled receptor; for details of the reported associations, see Supplementary Tables 2–5*.

Among the polymorphisms positively associated with PPI, 22 (26.2%) are functional variants, i.e., related to the level of gene expression or the biological function of the protein products (as reported in the reviewed studies). For the remaining 62 (73.8%) variants, no direct functional consequences were reported. For P50 gating, 4 (10.8%) polymorphisms positively associated with this measure are functional and 33 (89.2%) are without known functional consequences. To examine the potential functional role of the positively associated variants, we carried out an *in silico* analysis using the HaploReg resource (117). The results of this analysis showed that a substantial proportion of polymorphisms that were associated with PPI (41 SNPs) and P50 gating (14 SNPs) overlap with regulatory motifs such as promoter/enhancer histone marks or DNase I hypersensitive sites (for a detailed description see Supplementary Tables 2–5).

## Discussion

In this paper, we reviewed the available data on the relationship between genetic variability and sensory information filtering in humans. More specifically, we summarized, in a systematic manner, findings from genetic association studies published in peer-reviewed journals, examining the effect of common genetic variants on two well-established parameters of gating functions, PPI and P50/N100 ERP suppression (jointly referred to as P50 gating), deficits of which are considered as schizophrenia endophenotypes. We found that association with PPI or P50 gating was reported for variants located within or near 37 and 13 genes, respectively, which are involved in a variety of biological processes, mostly related to neurotransmission and neurodevelopment. However, most of the polymorphisms positively associated with PPI and/or P50 gating were examined in only one study or were not consistently replicated in other studies. According to our criteria for reliability (i.e., association confirmed in at least two independent samples and positive outcomes outnumbering negative results), only four polymorphisms within four genes for PPI (*CHRNA3* rs1317286, *COMT* rs4680, *HTR2A* rs6311, and *TCF4* rs9960767) and one polymorphism for P50 gating (*CHRNA7* rs67158670) can be considered as reliable or consistent across the studies. Two of them (*COMT* rs4680 and *TCF4* rs9960767) were identified as significantly associated with PPI also by Quednow et al. (47), who included 16 independent samples into a meta-analysis. Although a large number of reported associations were with non-coding polymorphisms, our analysis shows that a substantial proportion of them may play a role in gene expression by affecting the binding of transcription factors or chromatin remodeling. However, since enhancers may activate transcription of their target genes over considerable distances, up to hundreds or even thousands of kilobases, caution should be taken when making inferences about the functional connection between non-coding variants in these regions and target genes (118).

### Replication of Association Results

From the 35 associations that were tested in more than one study, only 10 polymorphisms were reported to be significantly associated with gating in two or more studies. Low statistical power in some studies could increase the probability of false-negative results and unsuccessful replications. Although we excluded studies whose quality was evaluated as poor according to the Q-Genie scoring system, yet in 12 of 63 studies that fulfilled the criteria to be included in this review, sample size was lower than 50. Furthermore, a considerable number of negative replication results (14 of 30) come from samples that differed in ethnicity compared to the initial studies reporting positive results. Notably, in addition to genetic diversity, difference in startle response and PPI across ethnic groups (119) could decrease the number of successful replications. On the other hand, the non-replications seem not to be due to diversity in stimulation parameters since these did not substantially differ between almost all studies that had yielded discrepant outcomes. In the light of considerable heritability of gating functions (31, 35–42), the low number of reliably assessed genetic associations clearly indicates that, despite the relatively large number of genetic studies, current knowledge on the genetic architecture of gating functions remains very limited. Next, we will focus our discussion on the variants/genes consistently associated with sensory and/or sensorimotor gating functions.

### Catechol-O-Methyltransferase

Catechol-o-methyltransferase (COMT) is an enzyme degrading catecholamines. A single nucleotide G-A substitution at codon 158 results in a change from valine to methionine (Val158Met) causing a missense mutation with a lower metabolic activity of the enzyme. This polymorphism significantly affects dopamine turnover in the prefrontal cortex (PFC, Val allele associated with reduced PFC dopamine levels), PFC activity, and executive functions in healthy humans [for review see e.g., (120, 121)]. Numerous studies reported *COMT* Val158Met polymorphism to be related with liability to schizophrenia and several other mental disorders, but a recent meta-analysis did not confirm a significant association with schizophrenia (122). The Val allele was associated with weaker PPI in six of seven studies included in our review. In agreement with these reports, a study by Giakoumaki et al. (63) has shown that administration of a COMT inhibitor tolcapone increased PPI in Val allele carriers. As highlighted by the meta-analysis by Quednow et al. (47), the association of PPI with *COMT* Val158Met polymorphism is stronger in men than in women. Interestingly, a similar pattern of sex-dependent effects of this variation was also reported for response inhibition and linked with the activity of the PFC [(123), see also (124)]. Given the putative role of the PFC in the modulation of sensorimotor gating (125–128), it could be speculated that the prefrontal circuitry is also involved in the sex-specific effects of *COMT* genotype on PPI, which remains to be established in future studies. The evidence for the association of *COMT* Val158Met with P50 gating is less consistent, as a significant association was reported in seven and non-significant in nine studied samples.

Another *COMT* polymorphism, rs165599, has not fulfilled our reliability criteria but was reported to be significantly associated with both PPI and P50 gating (only in one study each). Functional consequences of this variation are less clear, although there is evidence indicating its relationship with COMT mRNA levels in the brain of healthy humans, and IQ and the presence of psychotic symptoms in patients with 22q11 deletion syndrome (129, 130). A large case-control study reported its association with schizophrenia in women but not in men, suggesting that this SNP confers a sex-specific genetic component in schizophrenia (131). Notably, rs165599 and rs4680 are both part of a three-marker haplotype (together with rs2075507) that has been implicated in COMT protein level, PFC function in obsessive–compulsive disorder and attention-deficit hyperactivity disorder (129, 132, 133). This haplotype was significantly associated with P50 in a sample of patients with bipolar disorder (but not in healthy controls) (25). Its relationship with PPI has not been studied yet, as far as we are informed.

To sum up, there is considerable evidence that genetic variability of *COMT* affects gating functions, which fits with the proposed role of dopamine in the PFC (134). Interestingly, however, disruption of PPI following administration of dopamine agonists in rodents has been attributed to modulation of striatal rather than cortical circuitry (135, 136). In humans, on the other hand, the effects of dopamine agonists on PPI are less evident and reliable (137). Given the importance of PPI to study the neurobiology of schizophrenia in animal models, it would be desirable in future studies to shed more light on the specific roles of dopamine in cortical and striatal processing related to gating in humans and rodents.

### Serotonin 2A Receptor

The *HTR2A* gene encodes a G-protein-coupled serotonin 2A receptor (5-HT2AR). In humans, 5-HT2AR is widely expressed throughout the brain with particularly high density in the neocortex (138). 5-HT2AR has been implicated in multiple brain functions such as learning, memory, and cognition [for review see (139)]. Importantly, several lines of evidence implicate 5-HT2AR in the pathophysiology of psychiatric disorders. First, genetic variants in the *HTR2A* gene and functional abnormalities of 5-HT2AR are associated with many psychiatric disorders including schizophrenia [for review see (140)]. Second, 5-HT2AR antagonists produce antipsychotic and antidepressant-like effects, whereas agonists have psychotomimetic properties including PPI-disruptive effects (140, 141). *HTR2A* rs6311 (also known as −1438A/G) is a functional SNP, which lies upstream of the *HTR2A* promoter region and alters its activity (142). Meta-analyses confirmed the association of this polymorphism with schizophrenia and obsessive–compulsive disorder (143–145). Given the involvement of serotonin in multiple neurobiological processes, warranted are further studies of the role of 5-HT2AR in gating and its relationship with schizophrenia.

### Nicotinic Acetylcholine Receptor

A lot of research implicates signaling via nicotinic acetylcholine receptor (nAChR) in gating, schizophrenia, and nicotine dependence [for review see (146)]. It is well established in rodents and humans that the agonist of nAChR nicotine enhances PPI and P50 gating [for review see (147)]. In humans, sensorimotor gating efficiency was found to be inversely related to nicotine dependence (148). Smoking and nicotine dependence are highly prevalent in schizophrenia, and it has been proposed that tobacco is used by the patients as self-medication to alleviate the symptoms, in particular the impairment of cognitive functions (149). Moreover, recent research indicates that nicotine dependence and schizophrenia may share a part of their genetic liability [for review see (150)]. Across the reviewed studies, consistent associations were reported between PPI and variation in *CHRNA3* gene as well as between P50 gating and *CHRFAM7A* gene.

*CHRNA3* is a part of a *CHRNA5–CHRNA3–CHRNB4* gene cluster on chromosome 15 (15q25 region), encoding α5, α3, and β4 subunits of the nAChR, linked in previous studies to nicotine dependence as well as schizophrenia (151–153). Our search specifically points to *CHRNA3* rs1317286, which was reported to be associated with nicotine dependence in a GWAS (154). The analysis using HaploReg indicates that this SNP overlaps with enhancer histone marks and may thus play a role in *CHRNA3* transcription. However, due to high linkage disequilibrium, it is difficult to determine causative variants in the *CHRNA5–CHRNA3–CHRNB4* cluster, which is under complex and coordinated regulatory control (155). Interestingly, TCF4 (see below) has been identified as one of the regulators of gene expression at this locus (156).

*CHRFAM7A* is a partial duplication of a gene encoding α7 nAChR, *CHRNA7*. Translation of CHRFAM7A is low, but it seems to negatively regulate α7 nAChR function [for review see (157)]. The P50 gating-associated polymorphism of *CHRFAM7A* denoted as rs67158670 (or *CHRFAM7A*Δ*2bp*) is a 2-bp deletion in exon 6. This mutation causes a frameshift in translation, resulting in a truncated protein, which is even a more potent inhibitor of α7 nAChR (157). Reduced expression of *CHRNA7* was found in the frontal cortex of schizophrenia patients post-mortem (158) and smoking counteracts this deficit (159). In addition to the association with P50 gating, studies reported association of *CHRFAM7A*Δ*2bp* with schizophrenia, bipolar disorder, and episodic memory [for review see (157)]. The impact of *CHRFAM7A*Δ*2bp* on brain development is debated, and research in this direction could bring new discoveries of the pathomechanistic links between gating deficits and schizophrenia.

### Transcription Factor 4

The *TCF4* gene codes for a basic helix–loop–helix protein, transcription factor 4, which belongs to a subclass of transcriptional regulators termed E-proteins. E-proteins bind to a specific promoter element known as the Ephrussi-box (E-box) to regulate transcription of target genes in various tissues including the brain [for review see (160)]. Although the precise physiological function of TCF4 is not yet fully understood, a recent study demonstrated that binding sites for TCF4 are present in a large number of genes involved in nervous system development, ion transport, and signal transduction (156). Moreover, this study also showed that TCF4 binding sites are found in many susceptibility genes implicated in common neurodevelopmental disorders including schizophrenia and autism spectrum disorders. Notably, several SNPs in *TCF4* itself have been directly linked to schizophrenia, underscoring the possible role of this gene in schizophrenia pathogenesis (161). Our analysis points to a reliable association of *TCF4* rs9960767 with PPI. Notably, Quednow et al. (90) reported that the effect of this polymorphism on PPI is moderated by smoking behavior, which fits with the regulatory role of TCF4 on the *CHRNA5–CHRNA3–CHRNB4* cluster (156). The association of this variation with schizophrenia was confirmed at a meta-analytic and genome-wide level [for review see (162)]. *TCF4* rs9960767 is located within intron 3 of the *TCF4* gene and has no direct obvious functional consequences. Neither is there evidence of its linkage disequilibrium with other common non-synonymous polymorphisms or causal variants, which alter TCF4 mRNA expression in adult human brain (163). Williams et al. (163) suggested that rs9960767 may exert effects on *TCF4* expression in a developmental context. Our findings support this notion as the HaploReg analysis indicates that rs9960767 may affect putative binding sites of transcription factors Foxa and STAT in the brain germinal matrix, which plays a critical role during brain development. All these findings indicate that sensorimotor gating deficit is a constituent of the neurodevelopmental insult, which is assumed to play a crucial role in the pathogenesis of schizophrenia (164).

### Common Genetic Factors of Gating Functions

Four polymorphisms out of 45 variants studied so far were reported to be significantly associated with both PPI and P50 gating. Given our criteria of reliability, however, none of these associations was reliable for both measures. Evidence for common genetic mechanisms underlying both sensory and sensorimotor gating thus remains elusive. Although sensory and sensorimotor gating represent related concepts, the hallmark of which is inhibition, the relationship between PPI and P50 suppression is not fully understood. Correlation between the magnitude of PPI and P50 suppression seems weak since most studies found no significant relationship between the two measures (165–171). Furthermore, PPI primarily relies on the processing in the brainstem and the basal ganglia, which is modulated by the cerebral cortex (125–128, 135, 172–176), while the sources of P50 ERP and P50 suppression are thought to be localized predominantly in the hippocampus, the temporal and the frontal lobes (167, 177–179). Given the importance of PPI and P50 gating in psychiatry, further research is warranted to clarify the relationship between these two phenomena in more detail at both the cognitive/psychological and neurobiological levels.

## Conclusion

Our review identified a considerable number of genetic variants associated with PPI or P50 gating in previous studies. However, a critical evaluation of the reports shows associations of only five polymorphisms (four for PPI and one for P50 gating) as consistently replicated across the studies. From these, only two variants (*HTR2A* rs6311 and *TCF4* rs9960767, both associated with PPI) also show a reliable association with schizophrenia (meta-analytic or genome-wide evidence). Although deficits in sensory and sensorimotor gating are widely considered as important endophenotypes of schizophrenia, the evidence for the common genetic etiology of the impaired gating functions and schizophrenia thus remains limited, and further large-scale studies are warranted to advance our understanding of this complex problem.

## Data Availability Statement

The original contributions presented in the study are included in the article/Supplementary Materials. Further inquiries can be directed to the corresponding author.

## Author Contributions

IR provided the concept and design of the study. DB and RR collected and analyzed the data. RR, IR, and DB wrote and revised the manuscript. All authors contributed to the article and approved the submitted version.

## Conflict of Interest

The authors declare that the research was conducted in the absence of any commercial or financial relationships that could be construed as a potential conflict of interest.
