# Supplementary material for: Genetic Determinants of Gating Functions: Do We Get Closer to Understanding Schizophrenia Etiopathogenesis?
Source: Front Psychiatry. 2020 Nov 25;11:550225. doi: 10.3389/fpsyt.2020.550225 (PMC7723973; doi:10.3389/fpsyt.2020.550225)
Supplement: Supplementary file 1 [file Data_Sheet_1.docx]

Supplementary Material

1. **Supplementary Data**

**Supplementary Data 1**

Search strategy for PubMed

((“prepulse inhibition”[Title/Abstract] OR "pre-pulse inhibition"[Title/Abstract] OR "sensorimotor gating"[Title/Abstract] OR "sensory-motor gating"[Title/Abstract] OR "sensori-motor gating"[Title/Abstract] OR “startle”[Title/Abstract] OR reflex, startle[MeSH Terms] OR prepulse inhibition[MeSH Terms]

OR

(("P50"[Title/Abstract] OR "n100"[Title/Abstract] OR "p200"[Title/Abstract] OR "P 50"[Title/Abstract] OR "n 100"[Title/Abstract] OR "p 200"[Title/Abstract]) AND (("event related potential*"[Title/Abstract] OR "event-related potential*"[Title/Abstract] OR “evoked potential*”[Title/Abstract]) OR (Evoked Potentials[MeSH Terms] OR N1 Wave[MeSH Terms] OR P2 Wave[MeSH Terms] OR P50 Wave[MeSH Terms])))

OR

“sensory gating”[Title/Abstract] OR “double click”[Title/Abstract] OR “paired-click”[Title/Abstract] OR “pair-click”[Title/Abstract] OR “pair* click”[Title/Abstract] OR “dual click”[Title/Abstract] OR “paired stimulus”[Title/Abstract] OR “paired pulse”[Title/Abstract] OR “pair pulse”[Title/Abstract] OR “double pulse”[Title/Abstract])

AND

(gene[Title/Abstract] OR genes[Title/Abstract] OR genot*[Title/Abstract] OR genom*[Title/Abstract] OR genet*[Title/Abstract] OR polymorphism*[Title/Abstract] OR variant*[Title/Abstract] OR mutation*[Title/Abstract] OR haplotype*[Title/Abstract] OR genotype[MeSH Terms] OR polymorphism, genetic[MeSH Terms])

AND

(human*[Title/Abstract] OR man[Title/Abstract] OR woman[Title/Abstract] OR men[Title/Abstract] OR women[Title/Abstract] OR children[Title/Abstract] OR male*[Title/Abstract] OR female*[Title/Abstract] OR probands[Title/Abstract] OR volunteers[Title/Abstract] OR subjects[Title/Abstract] OR individuals[Title/Abstract] OR participants[Title/Abstract]))

NOT

(mouse[Title] OR mice[Title] OR rat[Title] OR rats[Title] OR drosophila[Title] OR zebrafish[Title] OR hyperekplexia[Title/Abstract]))

Search strategy for SCOPUS

(((TITLE-ABS-KEY (“prepulse inhibiton”) OR TITLE-ABS-KEY (“pre-pulse inhibition”) OR TITLE-ABS-KEY (“sensorimotor gating”) OR TITLE-ABS-KEY (“sensory-motor gating”) OR TITLE-ABS-KEY (“sensori-motor gating”) OR TITLE-ABS-KEY (startle) OR INDEXTERMS (reflex, startle) OR INDEXTERMS (prepulse inhibition)

OR

((TITLE-ABS-KEY (P50) OR TITLE-ABS-KEY (n100) OR TITLE-ABS-KEY (p200) OR TITLE-ABS-KEY (P 50) OR TITLE-ABS-KEY (n 100) OR TITLE-ABS-KEY (p 200)) AND ((TITLE-ABS-KEY (“event related potential*”) OR TITLE-ABS-KEY (“event-related potential*”) OR TITLE-ABS-KEY (“evoked potential*”)) OR (INDEXTERMS (Evoked Potentials) OR INDEXTERMS (N1 Wave) OR INDEXTERMS (P2 Wave) OR INDEXTERMS (P50 Wave))))

OR

TITLE-ABS-KEY (“sensory gating”) OR TITLE-ABS-KEY (“double click”) OR TITLE-ABS-KEY (“paired-click”) OR TITLE-ABS-KEY (“pair-click”) OR TITLE-ABS-KEY (“pair* click”) OR TITLE-ABS-KEY (“dual click”) OR TITLE-ABS-KEY (“paired stimulus”) OR TITLE-ABS-KEY (“paired pulse”) OR TITLE-ABS-KEY (“pair pulse”) OR TITLE-ABS-KEY (“double pulse”))

AND

(TITLE-ABS-KEY (gene) OR TITLE-ABS-KEY (genes) OR TITLE-ABS-KEY (genot*) OR TITLE-ABS-KEY (genom*) OR TITLE-ABS-KEY (genet*) OR TITLE-ABS-KEY (polymorphism) OR TITLE-ABS-KEY (variant*) OR TITLE-ABS-KEY (mutation*) OR TITLE-ABS-KEY (haplotype*) OR TITLE-ABS-KEY (genotype) OR INDEXTERMS (polymorphism, genetic))

AND

(TITLE-ABS-KEY (human*) OR TITLE-ABS-KEY (man) OR TITLE-ABS-KEY (woman) OR TITLE-ABS-KEY (men) OR TITLE-ABS-KEY (men) OR TITLE-ABS-KEY (women) OR TITLE-ABS-KEY (children) OR TITLE-ABS-KEY (male*) OR TITLE-ABS-KEY (female*) OR TITLE-ABS-KEY (probands) OR TITLE-ABS-KEY (volunteers) OR TITLE-ABS-KEY (subjects) OR TITLE-ABS-KEY (individuals) OR TITLE-ABS-KEY (participants)))

AND NOT

( TITLE ( mouse ) OR TITLE ( mice ) OR TITLE ( rat ) OR TITLE ( rats ) OR TITLE ( drosophila ) OR TITLE ( zebrafish ) OR TITLE ( hyperekplexia )))

# Supplementary Tables

**Supplementary Table 1**

Summary of the studies included in the review.

| **Study** | **Type of study** | **Q-Genie score (max 70)** | **Sample** | **Sample ID** | **Sample size** | **Race/ethnicity** | **Males %** | **Age** | **Outcomes** |
| --- | --- | --- | --- | --- | --- | --- | --- | --- | --- |
| Ancín et al. (2011) | CGAS | 47 | HS | 1 | 95 | European | 47.5 | 42.6 | P50 |
|  |  |  | BD | 2 | 122 | European | 36.9 | 44.8 | P50 |
| Bertelsen et al. (2015) | CGAS | 36 | SZ | 3 | 49 | European | 73.5 | 25.4 | PPI, P50 |
|  |  |  | HS | 4 | 106 | European | 71.7 | 26.7 | PPI, P50 |
| Bräuer et al. (2009) | CGAS | 44 | HS | 5 | 81 | European | 46.9 | 23.9 | PPI |
| Cabranes et al. (2013) | CGAS | 35 | HS | 1 | 95 | European | 47.4 | 42.4 | P50 |
|  |  |  | BD | 2 | 127 | European | 37.0 | 44.1 | P50 |
|  |  |  | SZ | 6 | 153 | European | 71.9 | 37.3 | P50 |
| Comasco et al. (2015) | CGAS | 42 | NAPW | 7 | 154 | European | 0 | 31.6 | PPI |
| Comasco et al. (2016) | CGAS | 40 | HPW | 7 | 128 | European | 0 | 32.2 | PPI |
| de Koning et al. (2012) | CGAS | 41 | DS | 8 | 23 | European | 48.0 | 29.5 | PPI |
| de Koning et al. (2015) | CGAS | 47 | DS | 8 | 23 | European | 48.0 | 29.5 | PPI |
| Demily et al. (2016) | CGAS | 38 | HS | 9 | 80 | European | n/a | n/a | P50 |
|  |  |  | SZ | 10 | 94 | European | n/a | n/a | P50 |
|  |  |  | HS,SZ | 9+10 | 174 | European | n/a | n/a | P50 |
| Flomen et al. (2013) | CGAS | 56 | PD, UR, HS | 11 | 445 | European | 46.1 | 39.9 | P50 |
| Gajewska et al. (2013) | pharmacogenetic study | 42 | HS | 12 | 114 | European | 50.0 | 26.6 | PPI |
| Giakoumaki et al. (2008) | pharmacogenetic study | 42 | HS | 13 | 12 (Val), 11 (Met) | European | 100.0 | 26.6 (Val); 24.6 ( Met) | PPI |
| Greenbaum et al. (2011) | CGAS | 34 | HS | 14 | 79 | Ashkenazi Jews | 33.0 | 23.9 | PPI |
| Greenwood et al. (2011) | CGAS | 54 | SZ, UR | 15 | 370 | diverse (European 89.0%; Hispanic, Native American, Asian and African American 11.0%) | n/a | n/a | PPI |
| Greenwood et al. (2012) | CGAS | 54 | HS, SZ | 16 | 219 | European | n/a | n/a | PPI |
|  |  |  | SZ | 17 | 76 | African American | n/a | n/a | PPI |
| Greenwood et al. (2019) | GWAS | 54 | HS | 15 | 321 | diverse (European >95%) | n/a | n/a | PPI |
|  |  |  | HS, SZ | 18 | 1212 | diverse (European 84.9%, Latino 15.1%) | n/a | n/a | PPI |
|  |  |  | HS, SZ | 15+18 | 1533 | Diverse (European >87.0, Latino) | 56.2 | 41.8 | PPI |
| Hall et al. (2014) | CGAS | 56 | HS | 19 | 74 | European | 43.2 | 35.8 | P50 |
|  |  |  | SZ, BD | 20 | 199 | European | 57.3 | 41.9 | P50 |
|  |  |  | HS, SZ, BD | 19+20 | 273 | European | 53.5 | 40.3 | P50 |
| Hall et al. (2015) | GWAS | 52 | HS, SZ, BD | 21 | 396 | European | 53.5 | n/a | P50 |
| Hashimoto et al. (2011) | CGAS | 46 | SZ | 22 | 53 | Japanese | 56.6 | 39.1 | PPI |
| Hessl et al. (2009) | CGAS | 38 | FXS, HS | 23 | 49 | diverse (European, African American, Native American, Hispanic) | 63.3 | 19.9 | PPI |
| Hokyo et al. (2010) | CGAS | 33 | SZ | 24 | 81 | Japanese | 54.3 | 53.8 | PPI |
|  |  |  | HS | 25 | 71 | Japanese | 40.8 | 46.4 | PPI |
| Hong et al. (2008) | CGAS | 44 | HS, SZ | 26+27+28+29 | 134 | diverse (European 54.0%, African American 36.5%, other 9.5%) | n/a | n/a | PPI |
|  |  |  | HS, SZ | 26+27 | n/a | European | n/a | n/a | PPI |
|  |  |  | HS, SZ | 28+29 | n/a | African American | n/a | n/a | PPI |
|  |  |  | HS | 26+28 | 44 | diverse (European 54.0%, African American 36.5%, other 9.5%) | 42.8 | 37.1 | PPI |
|  |  |  | SZ | 27+29 | 90 | diverse (European 55.8%, African American 41.6%, other 2.7%) | 72.6 | 39.1 | PPI |
|  |  |  | HS | 26 | n/a | European | n/a | n/a | PPI |
|  |  |  | SZ | 27 | n/a | European | n/a | n/a | PPI |
|  |  |  | HS | 28 | n/a | African American | n/a | n/a | PPI |
|  |  |  | SZ | 29 | n/a | African American | n/a | n/a | PPI |
| Kirenskaya et al. (2015) | CGAS | 32 | HS | 30 | 47 | European | 100.0 | n/a | PPI |
|  |  |  | SZ | 31 | 46 | European | 100.0 | n/a | PPI |
| Knott et al. (2010) | pharmacogenetic study | 32 | HS | 32 | 24 | n/a | 54.2 | n/a | P50 |
| Lennertz et al. (2012) | CGAS | 37 | SZ | 33 | 69 | European | 69.6 | 34.7 | PPI |
| Leonard et al. (2002) | CGAS | 33 | HS | 34 | 151 | diverse (European 61%, African American 34%, Hispanic 5%) | n/a | n/a | P50 |
| Levin et al. (2009) | CGAS | 45 | HS (males) | 35 | n/a | Jewish | 100.0 | n/a | PPI |
|  |  |  | HS (females) | 36 | n/a | Jewish | 0 | n/a | PPI |
|  |  |  | HS | 35+36 | 133 | Jewish | 33.6 | 21.1 | PPI |
| Liu et al. (2013) | CGAS | 40 | SZ | 37 | 88 | Han Chinese | 65.7 | 24.7 | PPI, P50 |
| Lu et al. (2007) | CGAS | 33 | HS | 38 | 25 | European | 76.0 | 45.0 | P50 |
|  |  |  | SZ | 39 | 42 | European | 83.3 | 42.0 | P50 |
|  |  |  | HS, SZ | 38+39 | 67 | European | 80.6 | 43.1 | P50 |
| Majic et al. (2011) | CGAS | 39 | HS | 40 | 282 | European | 51.4 | 41.2 | P50, N100 |
| Mao et al. (2016) | CGAS | 44 | HS | 41 | 165 | Han Chinese | n/a | n/a | P50 |
|  |  |  | SZ | 42 | 139 | Han Chinese | n/a | n/a | P50 |
|  |  |  | HS, SZ | 41+42 | 304 | Han Chinese | n/a | n/a | P50 |
| Matsuno et al. (2015) | CGAS | 37 | SZ | 43 | 121 | Japanese | 62.0 | 36.7 | PPI |
| Millar et al. (2011) | pharmacogenetic study | 33 | HS | 32 | 24 | n/a | 54.2 | n/a | P50 |
| Montag et al. (2008) | CGAS | 36 | HS | 44 | 96 | European | 0.0 | 22.1 | PPI |
| Notzon et al. (2017) | CGAS | 39 | HS | 45 | 82 | European | 47.6 | 25.2 | PPI |
| Perkins et al. (2008) | pharmacogenetic study | 42 | HS | 46 | 101 | European | 36.0 | n/a | PPI |
| Petrovsky et al. (2010) | CGAS | 46 | SZ | 33 | 68 | European | 69.1 | 34.7 | PPI |
|  |  |  | HS | 47 | 96 | European | 47.9 | 26.0 | PPI |
| Petrovsky et al. (2013) | pharmacogenetic study | 37 | HS | 48 | 52 | n/a | 59.6 | 28.6 | PPI |
| Quednow et al. (2008) | CGAS | 41 | SZ | 33 | 68 | European | 67.6 | 34.8 | PPI |
| Quednow et al. (2009) | CGAS | 41 | HS | 47 | 99 | European | 48.5 | 26.2 | PPI |
|  |  |  | HS (males) | 47 | n/a | European | n/a | n/a | PPI |
| Quednow et al. (2010) | CGAS | 41 | SZ | 33 | 68 | European | 67.6 | 34.8 | PPI |
| Quednow et al. (2011) | CGAS | 44 | HS | 47 | 98 | European | 49.0 | 26.1 | PPI |
|  |  |  | SZ | 33 | 105 | European | 61.9 | 31.9 | PPI |
| Quednow et al. (2012) | CGAS | 56 | HS | 49 | 1821 | European | 42.4 | 36.0 | P50 |
| Raux et al. (2002) | CGAS | 32 | HS | 50 | 77 | European | 48.5 | 39.0 | P50 |
|  |  |  | SZ | 51 | 70 | European | 72.9 | 38.0 | P50 |
|  |  |  | HS, SZ | 50+51 | 147 | European | 59.9 | 38.5 | P50 |
| Roussos et al. (2008a) | CGAS | 44 | HS | 52 | 101 | European | 100.0 | 26.0 | PPI |
| Roussos et al. (2008b) | CGAS | 44 | HS | 52 | 93 | European | 100.0 | 26.2 | PPI |
| Roussos et al. (2009a) | CGAS | 57 | HS | 52 | 217 | European | 100.0 | 26.1 | PPI |
| Roussos et al. (2009b) | pharmacogenetic study | 37 | HS | 53 | 25 | European | 100.0 | 24.6 | PPI |
| Roussos et al. (2011a) | CGAS | 51 | HS | 54 | 445 | European | 100.0 | 22.1 | PPI |
| Roussos et al. (2011b) | CGAS | 55 | HS | 54 | 445 | European | 100.0 | 22.1 | PPI |
| Roussos et al. (2011c) | CGAS | 55 | HS | 54 | 445 | European | 100.0 | n/a | PPI |
| Roussos et al. (2013) | CGAS | 45 | HS | 55 | 690 | European | 100.0 | 22.7 | PPI |
| Roussos et al. (2016) | GWAS | 54 | HS | 55 | 686 | European | 100.0 | 22.5 | PPI |
|  |  |  | HS | 56 | 306 | European | 100.0 | 21.3 | PPI |
|  |  |  | HS | 55+56 | 992 | European | 100.0 | n/a | PPI |
| Rovný et al. (2018) | CGAS | 45 | HS | 57 | 96 | European | 83.0 | 24.2 | PPI |
| Salle et al. (2013) | CGAS | 37 | HS | 58 | 57 | n/a | 100.0 | 22.4 | P50 |
| Shaikh et al. (2011) | CGAS | 49 | PD, UR, HS | 11 | 451 | European | 46.6 | 41.8 | P50 |
| Shi et al. (2016) | CGAS | 47 | HS | 41 | 62 | Han Chinese | n/a | 40.8 | PPI |
|  |  |  | SZ | 42 | 77 | Han Chinese | n/a | 44.7 | PPI |
| Schuhmacher et al. (2012) | CGAS | 53 | SZ | 33 | 68 | European | 67.6 | 34.8 | PPI |
| Vogt et al. (2016) | CGAS | 44 | HS | 49 | 1811 | European | 42.9 | n/a | P50 |
| Völter et al. (2012) | CGAS | 43 | HS | 59 | 101 | European | 30.7 | 24.9 | PPI |
|  |  |  | HS | 47 | 96 | European | 48.5 | 26.2 | PPI |
| Vorstman et al. (2009) | CGAS | 37 | DS, HS | 60 | 40 | n/a | 65.0 | 14.1 | PPI, P50 |
| Wonodi et al. (2014) | CGAS | 43 | HS | 61 | 66 | diverse (European 62.1%, African American 37.9%) | 56.8 | 39.2 | P50 |
|  |  |  | SZ | 62 | 118 | diverse (European 72.0%, African American 28.0%) | 51.3 | 41.0 | P50 |
|  |  |  | HS, SZ | 61+62 | 184 | diverse (European 68.5%, African American 31.5%) | 53.3 | 40.4 | P50 |
| Zarchi et al. (2013) | CGAS | 39 | DS | 63 | 41 | Jewish | 48.8 | 20.6 | P50 |

Notes: BD, patients with bipolar disorder; CGAS, candidate gene association study; DS, patients with 22q11 deletion syndrome; FXS, Fragile X syndrome; GWAS, genome-wide association study; HPW, healthy postpartum women; HS, healthy subjects; n/a, information not available; NAPW, non‑anxious pregnant women; PD, patients with psychotic disorder; SZ, schizophrenia patients; UR, unaffected relatives.

**Supplementary Table 2**

Summary of PPI-associated variants

| **Gene symbol (HGNC)** | **Polymorphism ID** | **Polymorphism type** | **Functional consequences** | **Functional annotation (dbSNP)** | **Overlap with regulatory motifs (Haploreg)** | **Number of samples (sig./ null)** | **Sample ID** | **ISI** | ***p*-value** |
| --- | --- | --- | --- | --- | --- | --- | --- | --- | --- |
| *ANKK1* | rs1800497 | SNP | Y | missense |  | 3/3 | 44 (HS) | L | 0.25 |
|  |  |  |  |  |  |  | 47 (HS) | S,M,L | **<0.01** |
|  |  |  |  |  |  |  | 52 (HS) | M,L | NS |
|  |  |  |  |  |  |  | 55+56 (HS) | S,M,L | NS |
|  |  |  |  |  |  |  | 59 (HS) | S | >0.5 |
|  |  |  |  |  |  |  |  | M | 0.06 |
|  |  |  |  |  |  |  |  | L | **<0.01** |
|  |  |  |  |  |  |  |  | S,M,L | **<0.05** |
|  |  |  |  |  |  |  | 47+59 (HS) | S,M,L | **<0.01** |
| *AUTS2* | rs4718984 | SNP | Y | intron | EHM, DHS | 1/2 | 55 (HS) | S,M,L | NS |
|  |  |  |  |  |  |  | 56 (HS) | S,M,L | NS |
|  |  |  |  |  |  |  | 55+56 (HS) | S,M,L | **3.49e-8** |
| *AVPR1a* | RS1 | STR (microsatellite repeat) | Y | promoter | n/a | 1/2 | 35 (HS) | S,M,L | NS |
|  |  |  |  |  |  |  | 36 (HS) | S,M,L | NS |
|  |  |  |  |  |  |  | 35+36 (HS) | S | **0.002** |
|  |  |  |  |  |  |  |  | M | NS |
|  |  |  |  |  |  |  |  | L | NS |
|  | RS3 | STR (microsatellite repeat) | Y | promoter | n/a | 1/0 | 35 (HS) | S | **0.029** |
|  |  |  |  |  |  |  |  | M | **0.018** |
|  |  |  |  |  |  |  |  | L | **0.009** |
|  |  |  |  |  |  |  | 36 (HS) | S | NS |
|  |  |  |  |  |  |  |  | M | **0.023** |
|  |  |  |  |  |  |  |  | L | NS |
|  |  |  |  |  |  |  | 35+36 (HS) | S | **0.041** |
|  |  |  |  |  |  |  |  | M | **0.006** |
|  |  |  |  |  |  |  |  | L | **0.008** |
| *CAMK2A* | rs1432832 | SNP | N | 3´downstream | PHM, EHM | 1/0 | 15 (SZ, UR) | M | **8.1e-4** |
| *CHRNA3* | rs1051730 | SNP | N | synonymous |  | 2/2 | 33 (SZ) | S | NS |
|  |  |  |  |  |  |  |  | M | NS |
|  |  |  |  |  |  |  |  | L | **0.02** |
|  |  |  |  |  |  |  | 47 (HS) | S,M,L | **<0.05** |
|  |  |  |  |  |  |  | 48 (HS) | S,M,L | 0.31 |
|  |  |  |  |  |  |  | 55+56 (HS) | S,M,L | NS |
|  | rs1317286 | SNP | N | intron |  | 2/1 | 33 (SZ) | S | NS |
|  |  |  |  |  |  |  |  | M | NS |
|  |  |  |  |  |  |  |  | L | **<0.05** |
|  |  |  |  |  |  |  | 47 (HS) | S,M,L | **0.02** |
|  |  |  |  |  |  |  | 55+56 (HS) | S,M,L | NS |
| *CHRNA4* | rs1044396 | SNP | N | missense | DHS | 1/1 | 41 (HS) | S,M,L | >0.05 |
|  |  |  |  |  |  |  | 42 (SZ) | S | >0.05 |
|  |  |  |  |  |  |  |  | M | >0.05 |
|  |  |  |  |  |  |  |  | L | **0.013** |
| *CHRNA7* | rs3826029 | SNP | N | 2KB upstream | PHM, DHS | 1/1 | 15 (SZ, UR) | M | **6.5e-4** |
|  |  |  |  |  |  |  | 55+56 (HS) | S,M,L | NS |
|  | rs885071 | SNP | N | intron | PHM | 1/1 | 15 (SZ, UR) | M | **5.7e-4** |
|  |  |  |  |  |  |  | 55+56 (HS) | S,M,L | NS |
|  | rs8035668 | SNP | N | intron | PHM, EHM | 1/0 | 17 (SZ) | M | **0.002** |
| *COMT* | rs4680 | SNP | Y | missense | EHM, DHS | 8/4 | 7 (NAPW) | L | **<0.05** (72 dB) |
|  |  |  |  |  |  |  |  | L | **<0.05** (74 dB) |
|  |  |  |  |  |  |  |  | L | > 0.05 (78 dB) |
|  |  |  |  |  |  |  |  | L | > 0.05 (86 dB) |
|  |  |  |  |  |  |  | 8 (DS) | S,L | **0.01** |
|  |  |  |  |  |  |  | 13 (HS) | S,M,L | **0.02** |
|  |  |  |  |  |  |  | 31 (SZ) | M,L | >0.05 |
|  |  |  |  |  |  |  | 30 (HS) | M | **<0.05** |
|  |  |  |  |  |  |  |  | L | **<0.05** |
|  |  |  |  |  |  |  | 33 (SZ) | S | NS |
|  |  |  |  |  |  |  |  | M | NS |
|  |  |  |  |  |  |  |  | L | **<0.05** |
|  |  |  |  |  |  |  | 37 (SZ) | L | >0.05 |
|  |  |  |  |  |  |  | 44 (HS) | L | 0.193 |
|  |  |  |  |  |  |  | 47 (HS- male) | S,M,L | **<0.05** |
|  |  |  |  |  |  |  | 52 (HS) | M,L | **0.001** |
|  |  |  |  |  |  |  | 55+56 (HS) | S,M,L | **0.01** |
|  |  |  |  |  |  |  | 60 (DS) | L | NS |
|  | rs174697 | SNP | N | intron | EHM, DHSs | 1/0 | 15 (SZ, UR) | M | **7.0e-4** |
|  | rs165599 | SNP | Y | 3´UTR | EHM, DHS | 1/0 | 37 (SZ) | L | **0.015** |
|  | rs4818 | SNP | Y | synonymous | EHM, DHS | 1/1 | 53 (HS) | S,M,L | **<0.05** (75, 85 dB) |
|  |  |  |  |  |  |  | 55+56 (HS) | S,M,L | 0.74 |
| *CTNNA2* | rs4852550 | SNP | N | intron | PHM | 1/0 | 16 (HS, SZ) | M | **0.009** |
| *DAO* | rs4623951 | SNP | N | 5´upstream |  | 1/0 | 54 (HS) | S | 0.13 (pooled), 0.9 (75dB), **0.015** (85 dB) |
|  |  |  |  |  |  |  |  | M | **0.004** (pooled), 0.07 (75 dB), **0.004** (85 dB) |
|  |  |  |  |  |  |  |  | L | 0.16 (pooled), 0.23 (75 dB), 0.2 (85 dB) |
| *DBH* | rs2073833 | SNP | N | 3´downstream | PHM, DHS | 1/0 | 15 (SZ, UR) | M | **3.2e-4** |
| *DRD3* | rs6280 | SNP | Y | promoter |  | 1/1 | 52 (HS) | S,M,L | **0.008** (75, 85 dB) |
|  |  |  |  |  |  |  | 55+56 (HS) | S,M,L | 0.62 |
| *ERBB4* | rs1521539 | SNP | N | intron |  | 1/0 | 17 (SZ) | M | **0.003** |
|  | rs1521545 | SNP | N | intron | EHM | 1/0 | 17 (SZ) | M | **0.008** |
|  | rs1546717 | SNP | N | intron | EHM, DHS | 1/0 | 17 (SZ) | M | **0.002** |
|  | rs9288446 | SNP | N | intron |  | 1/0 | 17 (SZ) | M | **0.008** |
| *FMR1* | n/a | CGG triplet repeat mutation in FMR1 at Xq27.3 | Y | promoter | n/a | 1/0 | 23 (FXS, HS) | M,L | **2.0e-6** |
| *GRID2* | rs1485015 | SNP | N | intron | DHS | 1/1 | 15 (SZ, UR) | M | **0.009** |
|  |  |  |  |  |  |  | 55+56 (HS) | S,M,L | 0.84 |
|  | rs1583337 | SNP | N | intron | EHM | 1/0 | 15 (SZ, UR) | M | **0.001** |
|  | rs1948016 | SNP | N | intron |  | 1/1 | 15 (SZ, UR) | M | **0.008** |
|  |  |  |  |  |  |  | 55+56 (HS) | S,M,L | 0.82 |
|  | rs2196320 | SNP | N | intron |  | 1/0 | 15 (SZ, UR) | M | **0.002** |
|  | rs2870699 | SNP | N | intron | DHS | 1/1 | 15 (SZ, UR) | M | **3.3e-4** |
|  |  |  |  |  |  |  | 55+56 (HS) | S,M,L | 0.24 |
|  | rs1039938 | SNP | N | intron |  | 1/0 | 17 (SZ) | M | **0.004** |
|  | rs6844474 | SNP | N | intron | EHM, DHS | 1/0 | 17 (SZ) | M | **0.007** |
|  | rs7662498 | SNP | N | intron |  | 1/0 | 16 (HS, SZ) | M | **6.0e-4** |
| *GRIK3* | rs575605 | SNP | N | intron | EHM, DHS | 1/1 | 15 (SZ, UR) | M | **0.01** |
|  |  |  |  |  |  |  | 55+56 (HS) | S,M,L | 0.45 |
|  | rs1027599 | SNP | N | intron |  | 1/1 | 16 (HS, SZ) | M | **0.001** |
|  |  |  |  |  |  |  | 55+56 (HS) | S,M,L | 0.15 |
|  | rs517176 | SNP | N | intron |  | 1/0 | 17 (SZ) | M | **0.003** |
|  | rs533337 | SNP | N | intron |  | 1/1 | 16 (HS, SZ) | M | **0.004** |
|  |  |  |  |  |  |  | 55+56 (HS) | S,M,L | 0.16 |
| *GRIN2A* | rs4782262 | SNP | N | intron |  | 1/0 | 16 (HS, SZ) | M | **0.006** |
| *GRIN3A* | rs3739724 | SNP | N | 3´UTR |  | 1/1 | 15 (SZ, UR) | M | **0.005** |
|  |  |  |  |  |  |  | 55+56 (HS) | S,M,L | 0.06 |
|  | rs942141 | SNP | N | intron |  | 1/1 | 15 (SZ, UR) | M | **0.004** |
|  |  |  |  |  |  |  | 55+56 (HS) | S,M,L | 0.47 |
| *GRIN3B* | rs10666583 | insertion | Y | frameshift | DHS | 1/0 | 43 (SZ) | L | **0.036** |
| *HTR2A* | rs6311 | SNP | Y | promoter | PHM, EHM, DHS | 2/1 | 33 (SZ) | L | **0.03** |
|  |  |  |  |  |  |  | 47 (HS) | S,M,L | **0.007** |
|  |  |  |  |  |  |  | 55+56 (HS) | S,M,L | 0.71 |
|  | rs6313 | SNP | Y | synonymous | PHM, EHM, DHS | 2/2 | 5 (HS) | M, L | >0.05 |
|  |  |  |  |  |  |  | 33 (SZ) | L | **0.03** |
|  |  |  |  |  |  |  | 47 (HS) | S,M,L | **0.007** |
|  |  |  |  |  |  |  | 55+56 (HS) | S,M,L | 0.71 |
| *KCNQ2* | rs3746372 | SNP | N | 3´UTR | DHS | 1/1 | 41 (HS) | S,M,L | >0.05 |
|  |  |  |  |  |  |  | 42 (SZ) | S | >0.05 |
|  |  |  |  |  |  |  |  | M | >0.05 |
|  |  |  |  |  |  |  |  | L | **0.035** |
| *KPNA4* | rs4130284 | SNP | Y | intron | EHM | 1/0 | 55 (HS) | S,M,L | **0.014** |
| *NCAM1* | rs1245119 | SNP | N | intron | PHM, EHM, DHS | 1/0 | 15 (SZ, UR) | M | **0.006** |
|  | rs1261878 | SNP | N | intron | EHM | 1/1 | 15 (SZ, UR) | M | **9.4e-4** |
|  |  |  |  |  |  |  | 55+56 (HS) | S,M,L | 0.41 |
|  | rs2196456 | SNP | N | intron | EHM | 1/0 | 15 (SZ, UR) | M | **0.004** |
| *NOS1* | Ex1f-VNTR | VNTR | Y | promoter | n/a | 1/0 | 57 (HS) | S | **0.016** |
|  |  |  |  |  |  |  |  | M | 0.152 |
|  |  |  |  |  |  |  |  | L | **0.026** |
|  |  |  |  |  |  |  |  | S,M,L | **0.003** |
|  | rs6490121 | SNP | Y | intron | DHS | 1/0 | 57 (HS) | S | **0.043** |
|  |  |  |  |  |  |  |  | M | 0.096 |
|  |  |  |  |  |  |  |  | L | 0.107 |
|  |  |  |  |  |  |  |  | S,M,L | **0.036** |
| *NOS1AP* | rs1538018 | SNP | N | intron |  | 1/0 | 15 (SZ, UR) | M | **0.009** |
| *NRG1* | rs4512342 | SNP | N | intron | EHM, DHS | 1/0 | 15 (SZ, UR) | M | **0.006** |
|  | rs7822564 | SNP | N | intron | DHS | 1/0 | 15 (SZ, UR) | M | **0.005** |
|  | rs901561 | SNP | N | intron | DHS | 1/1 | 15 (SZ, UR) | M | **0.009** |
|  |  |  |  |  |  |  | 55+56 (HS) | S,M,L | 0.42 |
|  | rs3924999 | SNP | N | missense |  | 6/6 | 26 (HS) | L | **0.03** |
|  |  |  |  |  |  |  | 27 (SZ) | L | **0.02** |
|  |  |  |  |  |  |  | 28 (HS) | L | NS |
|  |  |  |  |  |  |  | 29 (SZ) | L | NS |
|  |  |  |  |  |  |  | 28+29 (HS, SZ) | L | 0.35 |
|  |  |  |  |  |  |  | 26+27+28+29 (HS, SZ) | L | **0.003** |
|  |  |  |  |  |  |  | 26+27 (HS, SZ) | L | **0.01** |
|  |  |  |  |  |  |  | 26+28 (HS) | L | **0.02** |
|  |  |  |  |  |  |  | 27+29 (SZ) | L | **0.02** |
|  |  |  |  |  |  |  | 47 (HS) | S,M,L | 0.6 |
|  |  |  |  |  |  |  | 54 (HS) | S,M,L | NS |
|  |  |  |  |  |  |  | 55+56 (HS) | S,M,L | 0.76 |
|  | rs2439272 | SNP | N | intron | EHM | 1/0 | 54 (HS) | S | **0.04** (75 dB), **0.047** (85 dB) |
|  |  |  |  |  |  |  |  | M | 0.09 (75 dB), **3.0e-4** (85 dB) |
|  |  |  |  |  |  |  |  | L | **0.005** (75 dB), **0.047** (85 dB) |
|  |  |  |  |  |  |  |  | S,M,L | **0.002** (75 and 85 dB) |
|  | rs6994992 | SNP | Y | 5´upstream | DHS | 1/1 | 54 (HS) | S | **0.048** (75 dB), **0.01** (85 dB) |
|  |  |  |  |  |  |  |  | M | >0.5 (75 dB), **0.02** (85 dB) |
|  |  |  |  |  |  |  |  | L | >0.5 (75 dB), >0.1 (85 dB) |
|  |  |  |  |  |  |  |  | S,M,L | **0.02** |
|  |  |  |  |  |  |  | 55+56 (HS) | S,M,L | 0.97 |
|  | rs62510682 | SNP | Y | 5´upstream |  | 1/0 | 54 (HS) | S | **0.04** (75 dB), **0.02** (85 dB) |
|  |  |  |  |  |  |  |  | M | >0.5 (75 dB), >0.3 (85 dB) |
|  |  |  |  |  |  |  |  | L | >0.1 (75 dB), >0.9 (85 dB) |
|  |  |  |  |  |  |  |  | S,M,L | NS |
| *OXTR* | rs237885 | SNP | N | intron |  | 1/0 | 7 (HPW) | L | **<0.05** (72 dB) |
|  |  |  |  |  |  |  |  | L | >0.05 (74 dB) |
|  |  |  |  |  |  |  |  | L | **<0.05** (78 dB) |
|  |  |  |  |  |  |  |  | L | **<0.05** (86 dB) |
| *PRODH* | rs372055 | SNP | N | synonymous | PHM, EHM, DHS | 1/1 | 8 (DS) | S,L | 0.66 |
|  |  |  |  |  |  |  | 52 (HS) | S | 0.91 (75 dB), 0.07 (85 dB) |
|  |  |  |  |  |  |  |  | M | **0.034** (75 dB), **0.04** (85 dB) |
|  |  |  |  |  |  |  |  | L | **0.019** (75 dB), **0.006** (85 dB) |
|  |  |  |  |  |  |  |  | S,M,L | NS |
|  | rs385440 | SNP | N | intron | EHM, DHS | 1/0 | 52 (HS) | S | **0.043** (75 dB), **0.002** (85 dB) |
|  |  |  |  |  |  |  |  | M | **0.001** (75 dB), 0.08 (85 dB) |
|  |  |  |  |  |  |  |  | L | **2.0e-4** (75 dB), **0.003** (85 dB) |
|  |  |  |  |  |  |  |  | S,M,L | NS |
|  | rs450046 | SNP | Y | missense | EHM, DHS | 1/1 | 52 (HS) | S | 0.059 (75 dB), **0.002** (85 dB) |
|  |  |  |  |  |  |  |  | M | **0.003** (75 dB), 0.09 (85 dB) |
|  |  |  |  |  |  |  |  | L | **0.001** (75 dB), **0.01** (85 dB) |
|  |  |  |  |  |  |  |  | S,M,L | NS |
|  |  |  |  |  |  |  | 55+56 (HS) | S,M,L | 0.13 |
| *RELN* | rs7341475 | SNP | N | intron |  | 1/0 | 14 (HS) | S | 0.56 |
|  |  |  |  |  |  |  |  | M | 0.78 |
|  |  |  |  |  |  |  |  | L | **0.01** |
|  | rs39343 | SNP | N | intron |  | 1/0 | 17 (SZ) | M | **0.003** |
|  | rs11820062 | SNP | Y | intron | PHM, EHM, DHS | 1/0 | 22 (SZ) | L | **0.019** (82 dB) |
|  |  |  |  |  |  |  |  | L | 0.97 (86 dB) |
|  |  |  |  |  |  |  |  | L | 0.11 (90 dB) |
|  | rs2306365 | SNP | Y | intron | EHM | 1/0 | 22 (SZ) | L | 0.08 (82 dB) |
|  |  |  |  |  |  |  |  | L | **0.036** (86 dB) |
|  |  |  |  |  |  |  |  | L | **0.044** (90 dB) |
|  | rs7119750 | SNP | Y | intron | EHM, DHS | 1/0 | 22 (SZ) | L | 0.066 (82 dB) |
|  |  |  |  |  |  |  |  | L | **0.033** (86 dB) |
|  |  |  |  |  |  |  |  | L | **0.044** (90 dB) |
| *SLC1A2* | rs1923292 | SNP | N | intron | PHM, EHM | 1/0 | 16 (HS, SZ) | M | **0.006** |
| *SLC6A3* | rs40184 | SNP | N | intron |  | 1/0 | 16 (HS, SZ) | M | **0.005** |
| *TCF4* | rs9960767 | SNP | N | intron | EHM | 2/1 | 33 (SZ) | S | NS |
|  |  |  |  |  |  |  |  | M | NS |
|  |  |  |  |  |  |  |  | L | **<0.05** |
|  |  |  |  |  |  |  | 47 (HS) | S | **<0.01** |
|  |  |  |  |  |  |  |  | M | **<0.05** |
|  |  |  |  |  |  |  |  | L | **<0.001** |
|  |  |  |  |  |  |  | 55+56 (HS) | S,M,L | 0.93 |
| *TSPAN2/NGF* | rs61810702 | SNP | Y | 3´downstream |  | 1/2 | 55 (HS) | S,M,L | NS |
|  |  |  |  |  |  |  | 56 (HS) | S,M,L | NS |
|  |  |  |  |  |  |  | 55+56 (HS) | S,M,L | **1.4e-9** |

Notes: BD, patients with bipolar disorder; DHS, DNAse I hypersensitive sites; DS, patients with 22q11 deletion syndrome; EHM, enhancer histone marks; FXS, Fragile X syndrome; HPW, healthy postpartum women; HS, healthy subjects; ISI, interstimulus interval; n/a, information not available; L, ISI ≥ 100 ms; M, 50 ≤ ISI < 100 ms; NS, not statistically significant (*p* > 0.05); NAPW, non-anxious pregnant women; PD, patients with psychotic disorder; PHM, promoter histone marks; S, ISI < 50 ms; SNP, single nucleotide polymorphism; STR, short tandem repeat; SZ, schizophrenia patients; UR, unaffected relatives; VNTR, variable number of tandem repeats; a value in parentheses following a p-value denotes prepulse intensity for which the statistic applies.

**Supplementary Table 3**

Summary of P50-associated variants

| **Gene symbol (HGNC)** | **Polymorphism ID** | **Polymorphism type** | **Functional consequences** | **Functional annotation (dbSNP)** | **Overlap with regulatory motifs (Haploreg)** | **Number of samples (sig./ null)** | **Sample ID** | **P50 suppression index** | ***p*-value** |
| --- | --- | --- | --- | --- | --- | --- | --- | --- | --- |
| *ANKK1* | rs1800497 | SNP | Y | missense |  | 1/0 | 32 (HS) | p50 gating difference wave | **<0.02** |
| *CACNA1C* | rs4765905 | SNP | N | intron | EHM, DHS | 1/2 | 19 (HS) | rP50 | 0.193 |
|  |  |  |  |  |  |  | 20 (SZ, BD) | rP50 | **0.017** |
|  |  |  |  |  |  |  | 19+20 (HS, SZ, BD) | rP50 | 0.089 |
|  | rs4765913 | SNP | N | intron | EHM, DHS | 1/2 | 19 (HS) | rP50 | 0.193 |
|  |  |  |  |  |  |  | 20 (SZ, BD) | rP50 | 0.193 |
|  |  |  |  |  |  |  | 19+20 (HS, SZ, BD) | rP50 | **0.026** |
| *CHRFAM7A* | rs67158670 | deletion | N | frameshift |  | 3/1 | 11 (PD, UR, HS) | rP50 | **8.0e-4** |
|  |  |  |  |  |  |  | 50 (HS) | rP50 | **<0.03** |
|  |  |  |  |  |  |  | 51 (SZ) | rP50 | NS |
|  |  |  |  |  |  |  | 50+51 (HS, SZ) | rP50 | **<0.02** |
|  | CHRFAM7A CNV | CNV | N | frameshift |  | 1/0 | 11 (PD, UR, HS) | rP50 | **0.03** |
| *COMT* | rs4680 | SNP | Y | missense | EHM, DHS | 7/9 | 1 (HS) | rP50 | NS |
|  |  |  |  |  |  |  | 2 (BD) | rP50 | **0.046** |
|  |  |  |  |  |  |  | 9 (HS) | rP50 | **0.029** |
|  |  |  |  |  |  |  | 10 (SZ) | rP51 | 0.457 |
|  |  |  |  |  |  |  | 9+10 (HS, SZ) | rP52 | NS |
|  |  |  |  |  |  |  | 11 (PD, UR, HS) | rP50 | NS |
|  |  |  |  |  |  |  |  | dP50 | NS |
|  |  |  |  |  |  |  | 37 (SZ) | rP50 | >0.05 |
|  |  |  |  |  |  |  |  | dP50 | >0.05 |
|  |  |  |  |  |  |  | 38 (HS) | rP50 | NS |
|  |  |  |  |  |  |  |  | dP50 | NS |
|  |  |  |  |  |  |  | 39 (SZ) | rP50 | **0.03** |
|  |  |  |  |  |  |  |  | dP50 | NS |
|  |  |  |  |  |  |  | 38+39 (HS, SZ) | rP50 | **0.02** |
|  |  |  |  |  |  |  |  | dP50 | NS |
|  |  |  |  |  |  |  | 40 (HS) | rP50 | 0.687 |
|  |  |  |  |  |  |  |  | rN100 | **0.012** |
|  |  |  |  |  |  |  | 41+42 (HS, SZ) | rP50 | 0.62 |
|  |  |  |  |  |  |  |  | dP50 | 0.97 |
|  |  |  |  |  |  |  | 58 (HS) | rP50 | **0.012** |
|  |  |  |  |  |  |  | 60 (DS) | rP50 | NS |
|  |  |  |  |  |  |  | 63 (DS) | rP50 | **<0.05** |
|  | rs165599 | SNP | Y | 3´UTR | EHM, DHS | 1/2 | 1 (HS) | rP50 | NS |
|  |  |  |  |  |  |  | 2 (BD) | rP50 | **3.0e-5** |
|  |  |  |  |  |  |  | 37 (SZ) | rP50 | NS |
|  |  |  |  |  |  |  |  | %P50 | NS |
|  |  |  |  |  |  |  |  | dP50 | NS |
| *DISC1* | rs7551537 | SNP | N | intron | PHM, EHM, DHS | 1/0 | 15 (SZ, UR) | dP50 | **0.008** |
|  | rs821597 | SNP | N | intron | DHS | 1/0 | 15 (SZ, UR) | dP50 | **4.0e-4** |
|  | rs821662 | SNP | N | intron |  | 1/0 | 15 (SZ, UR) | dP50 | **0.003** |
|  | rs843979 | SNP | N | intron |  | 1/0 | 15 (SZ, UR) | dP50 | **0.005** |
| *ERBB4* | rs1394785 | SNP | N | intron | DHS | 1/0 | 15 (SZ, UR) | dP50 | **0.006** |
| *FLRT2* | chr14_85617703_I | insertion | N | intron | n/a | 1/0 | 21 (HS, SZ, BD) | rP50 | **7.1e-9** |
|  | rs10132223 | SNP | N | 5´upstream |  | 1/0 | 21 (HS, SZ, BD) | rP50 | **1.3e-9** |
|  | rs10135204 | SNP | N | 5´upstream |  | 1/0 | 21 (HS, SZ, BD) | rP50 | **1.1e-8** |
|  | rs10144282 | SNP | N | 5´upstream |  | 1/0 | 21 (HS, SZ, BD) | rP50 | **6.9e-9** |
|  | rs10147584 | SNP | N | 5´upstream |  | 1/0 | 21 (HS, SZ, BD) | rP50 | **5.9e-9** |
|  | rs10149105 | SNP | N | 5´upstream |  | 1/0 | 21 (HS, SZ, BD) | rP50 | **5.1e-9** |
|  | rs7157801 | SNP | N | 5´upstream |  | 1/0 | 21 (HS, SZ, BD) | rP50 | **1.4e-8** |
|  | rs7158211 | SNP | N | 5´upstream |  | 1/0 | 21 (HS, SZ, BD) | rP50 | **9.4e-9** |
|  | rs9652388 | SNP | N | 5´upstream |  | 1/0 | 21 (HS, SZ, BD) | rP50 | **8.1e-9** |
| *GRID2* | rs1155646 | SNP | N | intron | EHM, DHS | 1/0 | 15 (SZ, UR) | dP50 | **0.004** |
|  | rs1435477 | SNP | N | intron |  | 1/0 | 15 (SZ, UR) | dP50 | **0.002** |
|  | rs1875705 | SNP | N | intron |  | 1/0 | 15 (SZ, UR) | dP50 | **0.003** |
|  | rs1912718 | SNP | N | intron |  | 1/0 | 15 (SZ, UR) | dP50 | **0.008** |
|  | rs4524351 | SNP | N | intron | DHS | 1/0 | 15 (SZ, UR) | dP50 | **0.008** |
| *GRIK4* | rs7948804 | SNP | N | intron | EHM, DHS | 1/0 | 15 (SZ, UR) | dP50 | **0.007** |
|  | rs948028 | SNP | N | intron | EHM, DHS | 1/0 | 15 (SZ, UR) | dP50 | **0.004** |
| *GRM3* | rs10487055 | SNP | N | 3´downstream | DHS | 1/0 | 15 (SZ, UR) | dP50 | **2.0e-4** |
| *SLC6A3* | rs28363170 (40-bp VNTR) | VNTR | Y | 3´UTR | n/a | 1/0 | 32 (HS) | dP50 | **<0.04** |
| *TCF4* | rs10401120 | SNP | N | intron |  | 1/0 | 49 (HS) | rP50 | **0.001** |
|  |  |  |  |  |  |  |  | %P50 | **6.9e-5** |
|  |  |  |  |  |  |  |  | dP50 | **0.001** |
|  | rs17512836 | SNP | N | intron |  | 1/3 | 19 (HS) | rP50 | NS |
|  |  |  |  |  |  |  | 20 (SZ, BD) | rP50 | NS |
|  |  |  |  |  |  |  | 19+20 (HS, SZ, BD) | rP50 | NS |
|  |  |  |  |  |  |  | 49 (HS) | rP50 | NS |
|  |  |  |  |  |  |  |  | %P50 | **2.0e-4** |
|  |  |  |  |  |  |  |  | dP50 | NS |
|  | rs17597926 | SNP | N | intron | EHM, DHS | 1/0 | 49 (HS) | rP50 | **0.001** |
|  |  |  |  |  |  |  |  | %P50 | **1.4e-4** |
|  |  |  |  |  |  |  |  | dP50 | **0.001** |
|  | rs9960767 | SNP | N | intron | EHM | 1/3 | 19 (HS) | rP50 | NS |
|  |  |  |  |  |  |  | 20 (SZ, DS) | rP50 | NS |
|  |  |  |  |  |  |  | 19+20 (HS, SZ, BD) | rP50 | NS |
|  |  |  |  |  |  |  | 49 (HS) | rP50 | **0.001** |
|  |  |  |  |  |  |  |  | %P50 | **4.5e-5** |
|  |  |  |  |  |  |  |  | dP50 | **0.001** |

Notes: BD, patients with bipolar disorder; CNV, copy number variation; DHS, DNAse I hypersensitive sites; dP50, P50 difference score index of sensory gating; DS, patients with 22q11 deletion syndrome; EHM, enhancer histone marks; HS, healthy subjects; n/a, information not available; NS, not statistically significant (*p* > 0.05); PD, patients with psychotic disorder; PHM, promoter histone marks; rP50, P50 ratio index of sensory gating; SNP, single nucleotide polymorphism; SZ, schizophrenia patients; UR, unaffected relatives; VNTR, variable number of tandem repeats; %P50, P50 percentual index of sensory gating.

**Supplementary Table 4**

Summary of associated haplotypes and genetic interactions on PPI

| **Gene symbol (HGNC)** | **Polymorphism ID** | **Polymorphism type** | **Functional consequences for individual SNPs or haplotype** | **Overlap with regulatory motifs (Haploreg)** |  | **Sample ID** | **ISI** | ***p*-value** |
| --- | --- | --- | --- | --- | --- | --- | --- | --- |
| *HTR1A, HTR2A* | rs6295 x  rs6313 | SNP, interaction | Y (individual SNPs) | PHM, EHM PHM, EHM, DHS |  | 5 (HS) | S | **0.019** |
|  |  |  |  |  |  |  | M | **0.018** |
|  |  |  |  |  |  |  | L | 0.31 |
| *DAO* | rs4623951, rs2111902 | SNP, diplotype | N | EHM, DHS |  | 54 (HS) | S | 0.4 (pooled), 0.7 (75 dB), 0.2 (85 dB) |
|  |  |  |  |  |  |  | M | **0.007**, T-T: **0.007**, C-T: **0.003** (pooled), **0.033**, T-T: **0.005** (75 dB), **0.005**, C-T: **6.0e-4** (85 dB) |
|  |  |  |  |  |  |  | L | **0.027**, T-T: **0.016** (pooled), 0.15 (75 dB), **0.026**, T-T: **0.01** (85 dB) |
|  | rs4623951, rs3741775 | SNP, diplotype | N |  |  | 54 (HS) | S | **0.006**, C-G: **9.0e-4** (pooled), **0.004**, C-G: **0.016** (75 dB), **0.006**, T-G: **0.06**, C-G: **0.002** (85 dB) |
|  |  |  |  |  |  |  | M | **6.0e-5**, T-G: **0.003**, C-G: **1.0e-4** (pooled), **8.0e-4**, T-G: **0.008**, C-G: **0.002** (75dB), **0.001**, T-G: **0.02**, C-G: **9.0e-4** (85 dB) |
|  |  |  |  |  |  |  | L | **0.005**, C-G: **0.007** (pooled), **0.036**, C-G: **0.012** (75 dB), **0.009**, T-G: **0.04**, C-G: **0.02** (85 dB) |
|  | rs4623951, rs3825251 | SNP, diplotype | N |  |  | 54 (HS) | S | 0.4 (pooled), 0.9 (75 dB), 0.1 (85 dB) |
|  |  |  |  |  |  |  | M | **0.03**, C-T: **0.006** (pooled), 0.3 (75 dB), C-T: **0.004** (85 dB) |
|  |  |  |  |  |  |  | L | 0.6 (pooled), 0.5 (75 dB), 0.4 (85 dB) |
|  | rs4623951, rs3918346 | SNP, diplotype | N | EHM |  | 54 (HS) | S | 0.3 (pooled), 0.9 (75 dB), 0.07 (85 dB) |
|  |  |  |  |  |  |  | M | **0.011**, C-C: **0.003** (pooled), 0.1 (75 dB), **0.012**, C-C: **0.001** (85dB) |
|  |  |  |  |  |  |  | L | 0.09 (pooled), 0.3 (75 dB), 0.07 (85 dB) |
|  | rs2111902, rs3825251 | SNP, diplotype | N | EHM, DHS |  | 54 (HS) | S | 0.07 (pooled), 0.2 (75 dB), 0.2 (85 dB) |
|  |  |  |  |  |  |  | M | 0.8 (pooled), **0.03** (75 dB), 0.5 (85 dB) |
|  |  |  |  |  |  |  | L | 0.3 (pooled), 0.3 (75 dB), 0.3 (85 dB) |
|  | rs3918346, rs3825251 | SNP, diplotype | N | EHM |  | 54 (HS) | S | **0.05** (pooled), **0.02** (75 dB), 0.1 (85 dB) |
|  |  |  |  |  |  |  | M | 0.6 (pooled), 0.06 (75 dB), 0.7 (85 dB) |
|  |  |  |  |  |  |  | L | 0.1 (pooled), **0.019** (75 dB), 0.4 (85 dB) |
| *DRD2, ANKK1* | rs1800497 x rs7122454 | SNP, interaction | Y (individual SNPs) |  |  | 59 (HS) | S,M,L | **<0.05** |
| *NRG1* | rs2439272, rs10503929 | SNP, diplotype | N | EHM DHS |  | 54 (HS) | S | >0.1 (75 dB), **0.047** (85 dB) |
|  |  |  |  |  |  |  | M | 0.06 (75 dB), **0.004** (85 dB) |
|  |  |  |  |  |  |  | L | **0.04** (75 dB), >0.2 (85 dB) |
|  |  |  |  |  |  |  | S,M,L | **0.003** (75 and 85 dB) |
|  | rs3924999, rs2439272 | SNP, diplotype | N | EHM EHM |  | 54 (HS) | S | 0.076 (75 dB), >0.2 (85 dB) |
|  |  |  |  |  |  |  | M | >0.3 (75 dB), **2.8e-4** (85 dB) |
|  |  |  |  |  |  |  | L | **0.028** (75 dB), 0.07 (85 dB) |
|  |  |  |  |  |  |  | S,M,L | **0.005** (75 and 85 dB) |
|  | rs3924999, rs2439272, rs10503929 | SNP, diplotype | N | EHM EHM DHS |  | 54 (HS) | S | >0.1 (75 dB), 0.085 (85dB) |
|  |  |  |  |  |  |  | M | 0.1 (75 dB), **0.005** (85 dB) |
|  |  |  |  |  |  |  | L | >0.1 (75 dB) >0.3 (85 dB) |
|  |  |  |  |  |  |  | S,M,L | NS (75 and 85 dB) |
|  | rs6994992, rs73235619 (SNP8NRG221132) | SNP, diplotype | N | DHS |  | 54 (HS) | S | **0.037** (75 dB), >0.1 (85dB) |
|  |  |  |  |  |  |  | M | >0.9 (75 dB), >0.1 (85dB) |
|  |  |  |  |  |  |  | L | >0.7 (75 dB), >0.3 (85dB) |
|  |  |  |  |  |  |  | S,M,L | NS (75 and 85 dB) |
| *PRODH* | rs385440, rs372055,  rs450046 | SNP, haplotype (TAG) | N | EHM, DHS EHM, DHS EHM, DHS |  | 52 (HS) | S | 0.91 (75 dB), 0.07 (85 dB) |
|  |  |  |  |  |  |  | M | **0.03** (75 dB), **0.04** (85 dB) |
|  |  |  |  |  |  |  | L | **0.02** (75 dB), **0.006** (85 dB) |
|  | - | SNP, haplotype (CGA) | N |  |  | 52 (HS) | S | 0.06 (75 dB), **0.002** (85 dB) |
|  |  |  |  |  |  |  | M | **0.004** (75 dB), 0.09 (85 dB) |
|  |  |  |  |  |  |  | L | **0.001** (75 dB), **0.01** (85 dB) |
|  |  |  |  |  |  |  | S,M,L | **0.001** (75, 85 dB) |
| *TPH2* | rs4570625, rs4565946 | SNP, haplotype | Y | EHM, DHS |  | 33 (SZ) | S | T-T: NS |
|  |  |  |  |  |  |  | M | T-T: NS |
|  |  |  |  |  |  |  | L | T-T: **0.02** |
|  |  | SNP, haplotype | Y |  |  | 33 (SZ) | S | G-T: NS |

Notes: HS, healthy subjects; ISI, interstimulus interval; L, ISI ≥ 100 ms; M, 50 ≤ ISI < 100 ms; NS, not statistically significant (*p* > 0.05); S, ISI < 50 ms; SNP, single nucleotide polymorphism; SZ, schizophrenia patients; a value in parentheses following a p-value denotes prepulse intensity for which the statistic applies.

**Supplementary Table 5**

Summary of P50-associated haplotypes

| **Gene symbol (HGNC)** | **Polymorphism ID** | **Polymorphism type** | **Functional consequences for individual SNPs or haplotype** | **Overlap with regulatory motifs (Haploreg)** | **Sample ID** | **P50 suppression index calculation method** | ***p*-value** |
| --- | --- | --- | --- | --- | --- | --- | --- |
| *COMT* | rs2075507,  rs4680,  rs165599 | SNP, haplotype | Y | PHM, EHM, DHS  PHM, EHM, DHS  EHM, DHS | 2 (BD) | rP50 | **0.03** |
| *CHRNA7/CHRFAM7A* | rs67158670, CHRFAM7A CNV | deletion, CNV, combined genotype | N | n/a  n/a | 50 (HS) | rP50 | **<0.03** |
|  |  |  |  |  | 50 (HS+SZ) | rP50 | **<0.02** |
| *CHRNA7* | promoter allelic variants −143 /A  −178−G  −180G/C  −190+G  −191G/A  rs28531779  −241A/G  −46G/T  rs149637464  −92G/A  −93C/G  −172+CGGGGG | SNP, combined genotype | N | n/a  n/a  n/a  n/a  n/a  PHM, EHM, DHS  n/a  n/a  PHM, EHM, DHS  n/a  n/a  n/a | 34 (HS) | rP50 | **<1.0e-4** |

Notes: BD, patients with bipolar disorder; CNV, copy number variation; HS, healthy subjects; ISI, interstimulus interval; n/a, information not available; rP50, P50 ratio index of sensory gating; SNP, single nucleotide polymorphism; SZ, schizophrenia patients.

**References:**

Ancín I, Cabranes JA, Vázquez-Álvarez B, Santos JL, Sánchez-Morla E, García-Jiménez MÁ, Fernández C, Barabash A. Sensory gating deficit is associated with catechol-O-methyltransferase polymorphisms in bipolar disorder. *World J Biol Psychiatry* (2011) **12**:376–84. doi:10.3109/15622975.2011.552192

Bertelsen B, Oranje B, Melchior L, Fagerlund B, Werge TM, Mikkelsen JD, Tümer Z, Glenthøj BY. Association Study of CHRNA7 Promoter Variants with Sensory and Sensorimotor Gating in Schizophrenia Patients and Healthy Controls: A Danish Case–Control Study. *NeuroMolecular Med* (2015) **17**:423–430. doi:10.1007/s12017-015-8371-9

Bräuer D, Strobel A, Hensch T, Diers K, Lesch K-P, Brocke B. Genetic variation of serotonin receptor function affects prepulse inhibition of the startle. *J Neural Transm* (2009) **116**:607–613. doi:10.1007/s00702-009-0222-0

Cabranes JA, Ancín I, Santos JL, Sánchez-Morla E, García-Jiménez MÁ, López-Ibor JJ, Barabash A. No effect of polymorphisms in the non-duplicated region of the CHRNA7 gene on sensory gating P50 ratios in patients with schizophrenia and bipolar disorder. *Psychiatry Res* (2013) **205**:276–8. doi:10.1016/j.psychres.2012.08.015

Comasco E, Gulinello M, Hellgren C, Skalkidou A, Sylven S, Sundström-Poromaa I. Sleep duration, depression, and oxytocinergic genotype influence prepulse inhibition of the startle reflex in postpartum women. *Eur Neuropsychopharmacol* (2016) **26**:767–776. doi:10.1016/J.EURONEURO.2016.01.002

Comasco E, Hellgren C, Olivier J, Skalkidou A, Sundström Poromaa I. Supraphysiological hormonal status, anxiety disorders, and COMT Val/Val genotype are associated with reduced sensorimotor gating in women. *Psychoneuroendocrinology* (2015) **60**:217–223. doi:10.1016/j.psyneuen.2015.06.019

de Koning MB, Boot E, Bloemen OJN, van Duin EDA, Abel KM, de Haan L, Linszen DH, van Amelsvoort TAMJ. Startle reactivity and prepulse inhibition of the acoustic startle response are modulated by catechol-O-methyl-transferase Val(158) Met polymorphism in adults with 22q11 deletion syndrome. *J Psychopharmacol* (2012) **26**:1548–60. doi:10.1177/0269881112456610

de Koning MB, van Duin EDA, Boot E, Bloemen OJN, Bakker JA, Abel KM, van Amelsvoort TAMJ. PRODH rs450046 and proline x COMT Val158Met interaction effects on intelligence and startle in adults with 22q11 deletion syndrome. *Psychopharmacology (Berl)* (2015) **232**:3111–3122. doi:10.1007/s00213-015-3971-5

de la Salle S, Smith D, Choueiry J, Impey D, Philippe T, Dort H, Millar A, Albert P, Knott V. Effects of COMT genotype on sensory gating and its modulation by nicotine: Differences in low and high P50 suppressors. *Neuroscience* (2013) **241**:147–156. doi:10.1016/j.neuroscience.2013.03.029

Demily C, Louchart-de-la-Chapelle S, Nkam I, Ramoz N, Denise P, Nicolas A, Savalle C, Thibaut F. Does COMT val158met polymorphism influence P50 sensory gating, eye tracking or saccadic inhibition dysfunctions in schizophrenia? *Psychiatry Res* (2016) **246**:738–744. doi:10.1016/j.psychres.2016.07.066

Flomen RH, Shaikh M, Walshe M, Schulze K, Hall M-H, Picchioni M, Rijsdijk F, Toulopoulou T, Kravariti E, Murray RM, et al. Association between the 2-bp deletion polymorphism in the duplicated version of the alpha7 nicotinic receptor gene and P50 sensory gating. *Eur J Hum Genet* (2013) **21**:76–81. doi:10.1038/ejhg.2012.81

Gajewska A, Blumenthal TD, Winter B, Herrmann MJ, Conzelmann A, Mühlberger A, Warrings B, Jacob C, Arolt V, Reif A, et al. Effects of ADORA2A gene variation and caffeine on prepulse inhibition: A multi-level risk model of anxiety. *Prog Neuro-Psychopharmacology Biol Psychiatry* (2013) **40**:115–121. doi:10.1016/j.pnpbp.2012.08.008

Giakoumaki SG, Roussos P, Bitsios P. Improvement of prepulse inhibition and executive function by the COMT inhibitor tolcapone depends on COMT Val158Met polymorphism. *Neuropsychopharmacology* (2008) **33**:3058–68. doi:10.1038/npp.2008.82

Greenbaum L, Levin R, Lerer E, Alkelai A, Kohn Y, Heresco-Levy U, Ebstein RP, Lerer B. Association of Reelin (RELN) single nucleotide polymorphism rs7341475 with prepulse inhibition in the Jewish Israeli population. *Biol Psychiatry* (2011) **69**:e17–e18. doi:10.1016/j.biopsych.2010.09.059

Greenwood TA, Lazzeroni LC, Maihofer AX, Swerdlow NR, Calkins ME, Freedman R, Green MF, Light GA, Nievergelt CM, Nuechterlein KH, et al. Genome-wide Association of Endophenotypes for Schizophrenia From the Consortium on the Genetics of Schizophrenia (COGS) Study. *JAMA Psychiatry* (2019) **76**:1274. doi:10.1001/jamapsychiatry.2019.2850

Greenwood TA, Lazzeroni LC, Murray SS, Cadenhead KS, Calkins ME, Dobie DJ, Green MF, Gur RE, Gur RC, Hardiman G, et al. Analysis of 94 candidate genes and 12 endophenotypes for schizophrenia from the Consortium on the Genetics of Schizophrenia. *Am J Psychiatry* (2011) **168**:930–46. doi:10.1176/appi.ajp.2011.10050723

Greenwood TA, Light GA, Swerdlow NR, Radant AD, Braff DL. Association analysis of 94 candidate genes and schizophrenia-related endophenotypes. *PLoS One* (2012) **7**:e29630. doi:10.1371/journal.pone.0029630

Hall M-H, Chen C-Y, Cohen BM, Spencer KM, Levy DL, Öngür D, Smoller JW. Genomewide association analyses of electrophysiological endophenotypes for schizophrenia and psychotic bipolar disorders: A preliminary report. *Am J Med Genet Part B Neuropsychiatr Genet* (2015) **168**:151–161. doi:10.1002/ajmg.b.32298

Hall M-H, Levy DL, Salisbury DF, Haddad S, Gallagher P, Lohan M, Cohen B, Öngür D, Smoller JW. Neurophysiologic effect of GWAS derived schizophrenia and bipolar risk variants. *Am J Med Genet Part B Neuropsychiatr Genet* (2014) **165**:9–18. doi:10.1002/ajmg.b.32212

Hashimoto R, Ohi K, Yasuda Y, Fukumoto M, Yamamori H, Takahashi H, Iwase M, Okochi T, Kazui H, Saitoh O, et al. Variants of the RELA Gene are Associated with Schizophrenia and their Startle Responses. *Neuropsychopharmacology* (2011) **36**:1921–1931. doi:10.1038/npp.2011.78

Hessl D, Berry-Kravis E, Cordeiro L, Yuhas J, Ornitz EM, Campbell A, Chruscinski E, Hervey C, Long JM, Hagerman RJ. Prepulse inhibition in fragile X syndrome: feasibility, reliability, and implications for treatment. *Am J Med Genet B Neuropsychiatr Genet* (2009) **150B**:545–53. doi:10.1002/ajmg.b.30858

Hokyo A, Kanazawa T, Uenishi H, Tsutsumi A, Kawashige S, Kikuyama H, Glatt SJ, Koh J, Nishimoto Y, Matsumura H, et al. Habituation in prepulse inhibition is affected by a polymorphism on the NMDA receptor 2B subunit gene (GRIN2B). *Psychiatr Genet* (2010) **20**:191–198. doi:10.1097/YPG.0b013e32833a201d

Hong LE, Wonodi I, Stine OC, Mitchell BD, Thaker GK. Evidence of missense mutations on the neuregulin 1 gene affecting function of prepulse inhibition. *Biol Psychiatry* (2008) **63**:17–23. doi:10.1016/j.biopsych.2007.05.011

Kirenskaya A V., Storozheva ZI, Kolobov V V., Sherstnev V V. The acoustic startle response and polymorphism of the gene for catechol-O-methyltransferase in the norm and in schizophrenia. *Neurochem J* (2015) **9**:76–83. doi:10.1134/S1819712415010031

Knott V, Millar A, Fisher D, Albert P. Effects of nicotine on the amplitude and gating of the auditory P50 and its influence by dopamine D2 receptor gene polymorphism. *Neuroscience* (2010) **166**:145–56. doi:10.1016/j.neuroscience.2009.11.053

Lennertz L, Quednow BB, Schuhmacher A, Petrovsky N, Frommann I, Schulze-Rauschenbach S, Landsberg MW, Steinbrecher A, Höfels S, Pukrop R, et al. The functional coding variant Asn107Ile of the neuropeptide S receptor gene (NPSR1) is associated with schizophrenia and modulates verbal memory and the acoustic startle response. *Int J Neuropsychopharmacol* (2012) **15**:1205–1215. doi:10.1017/S1461145711001623

Leonard S, Gault J, Hopkins J, Logel J, Vianzon R, Short M, Drebing C, Berger R, Venn D, Sirota P, et al. Association of promoter variants in the α7 nicotinic acetylcholine receptor subunit gene with an inhibitory deficit found in schizophrenia. *Arch Gen Psychiatry* (2002) **59**:1085–1096. doi:10.1001/archpsyc.59.12.1085

Levin R, Heresco-Levy U, Bachner-Melman R, Israel S, Shalev I, Ebstein RP. Association between arginine vasopressin 1a receptor (AVPR1a) promoter region polymorphisms and prepulse inhibition. *Psychoneuroendocrinology* (2009) **34**:901–8. doi:10.1016/j.psyneuen.2008.12.014

Liu X, Hong X, Chan RCK, Kong F, Peng Z, Wan X, Wang C, Cheng L. Association study of polymorphisms in the alpha 7 nicotinic acetylcholine receptor subunit and catechol-o-methyl transferase genes with sensory gating in first-episode schizophrenia. *Psychiatry Res* (2013) **209**:431–8. doi:10.1016/j.psychres.2013.03.027

Lu BY, Martin KE, Edgar JC, Smith AK, Lewis SF, Escamilla MA, Miller GA, Cañive JM. Effect of catechol O-methyltransferase val(158)met polymorphism on the p50 gating endophenotype in schizophrenia. *Biol Psychiatry* (2007) **62**:822–5. doi:10.1016/j.biopsych.2006.11.030

Majic T, Rentzsch J, Gudlowski Y, Ehrlich S, Juckel G, Sander T, Lang UE, Winterer G, Gallinat J. COMT Val108/158Met genotype modulates human sensory gating. *Neuroimage* (2011) **55**:818–824. doi:10.1016/j.neuroimage.2010.12.031

Mao Q, Tan Y-L, Luo X-G, Tian L, Wang Z-R, Tan S-P, Chen S, Yang G-G, An H-M, Yang F-D, et al. Association of catechol-O-methyltransferase Val 108/158 Met genetic polymorphism with schizophrenia, P50 sensory gating, and negative symptoms in a Chinese population. *Psychiatry Res* (2016) **242**:271–276. doi:10.1016/j.psychres.2016.04.029

Matsuno H, Ohi K, Hashimoto R, Yamamori H, Yasuda Y, Fujimoto M, Yano-Umeda S, Saneyoshi T, Takeda M, Hayashi Y. A Naturally Occurring Null Variant of the NMDA Type Glutamate Receptor NR3B Subunit Is a Risk Factor of Schizophrenia. *PLoS One* (2015) **10**:e0116319. doi:10.1371/journal.pone.0116319

Millar A, Smith D, Choueiry J, Fisher D, Albert P, Knott V. The moderating role of the dopamine transporter 1 gene on P50 sensory gating and its modulation by nicotine. *Neuroscience* (2011) **180**:148–56. doi:10.1016/j.neuroscience.2011.02.008

Montag C, Hartmann P, Merz M, Burk C, Reuter M. D2 receptor density and prepulse inhibition in humans: negative findings from a molecular genetic approach. *Behav Brain Res* (2008) **187**:428–32. doi:10.1016/j.bbr.2007.10.006

Notzon S, Vennewald N, Gajewska A, Klahn AL, Diemer J, Winter B, Fohrbeck I, Arolt V, Pauli P, Domschke K, et al. Is prepulse modification altered by continuous theta burst stimulation? DAT1 genotype and motor threshold interact on prepulse modification following brain stimulation. *Eur Arch Psychiatry Clin Neurosci* (2017) **267**:767–779. doi:10.1007/s00406-017-0786-x

Perkins KA, Lerman C, Coddington S, Jetton C, Karelitz JL, Wilson A, Jennings JR, Ferrell R, Bergen AW, Benowitz NL. Gene and gene by sex associations with initial sensitivity to nicotine in nonsmokers. *Behav Pharmacol* (2008) **19**:630–640. doi:10.1097/FBP.0b013e32830c3621

Petrovsky N, Ettinger U, Kessler H, Mössner R, Wolfsgruber S, Dahmen N, Maier W, Wagner M, Quednow BB. The effect of nicotine on sensorimotor gating is modulated by a CHRNA3 polymorphism. *Psychopharmacology (Berl)* (2013) **229**:31–40. doi:10.1007/s00213-013-3081-1

Petrovsky N, Quednow BB, Ettinger U, Schmechtig A, Mössner R, Collier D a, Kühn K-U, Maier W, Wagner M, Kumari V. Sensorimotor gating is associated with CHRNA3 polymorphisms in schizophrenia and healthy volunteers. *Neuropsychopharmacology* (2010) **35**:1429–1439. doi:10.1038/npp.2010.12

Quednow BB, Brinkmeyer J, Mobascher A, Nothnagel M, Musso F, Gründer G, Savary N, Petrovsky N, Frommann I, Lennertz L, et al. Schizophrenia risk polymorphisms in the TCF4 gene interact with smoking in the modulation of auditory sensory gating. *Proc Natl Acad Sci U S A* (2012) **109**:6271–6. doi:10.1073/pnas.1118051109

Quednow BB, Ettinger U, Mössner R, Rujescu D, Giegling I, Collier D a, Schmechtig A, Kühn K-U, Möller H-J, Maier W, et al. The schizophrenia risk allele C of the TCF4 rs9960767 polymorphism disrupts sensorimotor gating in schizophrenia spectrum and healthy volunteers. *J Neurosci* (2011) **31**:6684–6691. doi:10.1523/JNEUROSCI.0526-11.2011

Quednow BB, Kühn K-U, Mössner R, Schwab SG, Schuhmacher A, Maier W, Wagner M. Sensorimotor gating of schizophrenia patients is influenced by 5-HT2A receptor polymorphisms. *Biol Psychiatry* (2008) **64**:434–7. doi:10.1016/j.biopsych.2008.02.019

Quednow BB, Schmechtig A, Ettinger U, Petrovsky N, Collier D a., Vollenweider FX, Wagner M, Kumari V. Sensorimotor Gating Depends on Polymorphisms of the Serotonin-2A Receptor and Catechol-O-Methyltransferase, but Not on Neuregulin-1 Arg38Gln Genotype: A Replication Study. *Biol Psychiatry* (2009) **66**:614–620. doi:10.1016/j.biopsych.2009.05.007

Quednow BB, Wagner M, Mössner R, Maier W, Kühn K-UU. Sensorimotor gating of schizophrenia patients depends on catechol o-methyltransferase val158met polymorphism. *Schizophr Bull* (2010) **36**:341–346. doi:10.1093/schbul/sbn088

Raux G, Bonnet-Brilhault F, Louchart S, Houy E, Gantier R, Levillain D, Allio G, Haouzir S, Petit M, Martinez M, et al. The -2 bp deletion in exon 6 of the “alpha 7-like” nicotinic receptor subunit gene is a risk factor for the P50 sensory gating deficit. *Mol Psychiatry* (2002) **7**:1006–1011. doi:10.1038/sj.mp.4001140

Roussos P, Giakoumaki SG, Adamaki E, Bitsios P. The influence of schizophrenia-related neuregulin-1 polymorphisms on sensorimotor gating in healthy males. *Biol Psychiatry* (2011a) **69**:479–86. doi:10.1016/j.biopsych.2010.09.009

Roussos P, Giakoumaki SG, Adamaki E, Georgakopoulos A, Anastasios G, Robakis NK, Nikos RK, Bitsios P. The association of schizophrenia risk D-amino acid oxidase polymorphisms with sensorimotor gating, working memory and personality in healthy males. *Neuropsychopharmacology* (2011b) **36**:1677–1688. doi:10.1038/npp.2011.49

Roussos P, Giakoumaki SG, Bitsios P. A Risk PRODH Haplotype Affects Sensorimotor Gating, Memory, Schizotypy, and Anxiety in Healthy Male Subjects. *Biol Psychiatry* (2009a) **65**:1063–1070. doi:10.1016/j.biopsych.2009.01.003

Roussos P, Giakoumaki SG, Bitsios P. The Dopamine D3 Receptor Ser9Gly Polymorphism Modulates Prepulse Inhibition of the Acoustic Startle Reflex. *Biol Psychiatry* (2008a) **64**:235–240. doi:10.1016/j.biopsych.2008.01.020

Roussos P, Giakoumaki SG, Bitsios P. Tolcapone effects on gating, working memory, and mood interact with the synonymous catechol-O-methyltransferase rs4818c/g polymorphism. *Biol Psychiatry* (2009b) **66**:997–1004. doi:10.1016/j.biopsych.2009.07.008

Roussos P, Giakoumaki SG, Georgakopoulos A, Robakis NK, Bitsios P. The CACNA1C and ANK3 risk alleles impact on affective personality traits and startle reactivity but not on cognition or gating in healthy males. *Bipolar Disord* (2011c) **13**:250–259. doi:10.1111/j.1399-5618.2011.00924.x

Roussos P, Giakoumaki SG, Rogdaki M, Pavlakis S, Frangou S, Bitsios P. Prepulse inhibition of the startle reflex depends on the catechol O-methyltransferase Val158Met gene polymorphism. *Psychol Med* (2008b) **38**:1651–1658. doi:10.1017/S0033291708002912

Roussos P, Giakoumaki SG, Zouraraki C, Fullard JF, Karagiorga VE, Tsapakis EM, Petraki Z, Siever LJ, Lencz T, Malhotra A, et al. The Relationship of Common Risk Variants and Polygenic Risk for Schizophrenia to Sensorimotor Gating. *Biol Psychiatry* (2016) **79**:988–996. doi:10.1016/j.biopsych.2015.06.019

Roussos P, Katsel P, Davis KL, Giakoumaki SG, Lencz T, Malhotra AK, Siever LJ, Bitsios P, Haroutunian V. Convergent findings for abnormalities of the NF-κB signaling pathway in schizophrenia. *Neuropsychopharmacology* (2013) **38**:533–9. doi:10.1038/npp.2012.215

Rovný R, Marko M, Katina S, Murínová J, Roháriková V, Cimrová B, Repiská G, Minárik G, Riečanský I. Association between genetic variability of neuronal nitric oxide synthase and sensorimotor gating in humans. *Nitric Oxide* (2018) **80**:32–36. doi:10.1016/J.NIOX.2018.08.002

Schuhmacher A, Becker T, Rujescu D, Quednow BB, Lennertz L, Wagner M, Benninghoff J, Rietschel M, Häfner H, Franke P, et al. Investigation of tryptophan hydroxylase 2 (TPH2) in schizophrenia and in the response to antipsychotics. *J Psychiatr Res* (2012) **46**:1073–1080. doi:10.1016/j.jpsychires.2012.04.021

Shaikh M, Hall M-H, Schulze K, Dutt A, Walshe M, Williams I, Constante M, Picchioni M, Toulopoulou T, Collier D, et al. Do COMT, BDNF and NRG1 polymorphisms influence P50 sensory gating in psychosis? *Psychol Med* (2011) **41**:263–76. doi:10.1017/S003329170999239X

Shi J, Wang Z, Tan Y, Fan H, An H, Zuo L, Yang F, Tan S, Li J, Zhang X, et al. CHRNA4 was associated with prepulse inhibition of schizophrenia in Chinese: a pilot study. *Cogn Neuropsychiatry* (2016) **21**:156–167. doi:10.1080/13546805.2016.1155437

Vogt J, Yang J, Mobascher A, Cheng J, Li Y, Liu X, Baumgart J, Thalman C, Kirischuk S, Unichenko P, et al. Molecular cause and functional impact of altered synaptic lipid signaling due to a prg‐1 gene SNP. *EMBO Mol Med* (2016) **8**:25–38. doi:10.15252/emmm.201505677

Völter C, Riedel M, Wöstmann N, Aichert DS, Lobo S, Costa A, Schmechtig A, Collier DA, Hartmann AM, Giegling I, et al. Sensorimotor gating and D2 receptor signalling: evidence from a molecular genetic approach. *Int J Neuropsychopharmacol* (2012) **15**:1427–1440. doi:10.1017/S1461145711001787

Vorstman JAS, Turetsky BI, Sijmens-Morcus MEJ, de Sain MG, Dorland B, Sprong M, Rappaport EF, Beemer FA, Emanuel BS, Kahn RS, et al. Proline Affects Brain Function in 22q11DS Children with the Low Activity COMT158 Allele. *Neuropsychopharmacology* (2009) **34**:739–746. doi:10.1038/npp.2008.132

Wonodi I, McMahon RP, Krishna N, Mitchell BD, Liu J, Glassman M, Elliot Hong L, Gold JM. Influence of kynurenine 3-monooxygenase (KMO) gene polymorphism on cognitive function in schizophrenia. *Schizophr Res* (2014) **160**:80–87. doi:10.1016/j.schres.2014.10.026

Zarchi O, Carmel M, Avni C, Attias J, Frisch A, Michaelovsky E, Patya M, Green T, Weinberger R, Weizman A, et al. Schizophrenia-like neurophysiological abnormalities in 22q11.2 deletion syndrome and their association to COMT and PRODH genotypes. *J Psychiatr Res* (2013) **47**:1623–1629. doi:10.1016/j.jpsychires.2013.07.004
